# Cullin-7 (CUL7) is overexpressed in glioma cells and promotes tumorigenesis via NF-κB activation

**DOI:** 10.1186/s13046-020-01553-7

**Published:** 2020-04-06

**Authors:** Jianye Xu, Zongpu Zhang, Mingyu Qian, Shaobo Wang, Wei Qiu, Zihang Chen, Zhongzheng Sun, Ye Xiong, Chaochao Wang, Xiaopeng Sun, Rongrong Zhao, Hao Xue, Gang Li

**Affiliations:** 1 Department of Neurosurgery, Qilu Hospital of Shandong University, 107 Wenhua Xi Road, Jinan, 250012 Shandong Province China; 2 Institute of Brain and Brain-Inspired Science, Shandong University, 107 Wenhua Xi Road, Jinan, 250012 Shandong Province China; 3 Shandong Key Laboratory of Brain Function Remodeling, Shandong University, 107 Wenhua Xi Road, Jinan, 250012 Shandong Province China; 4 Department of Neurosurgery, The Second Hospital of Shandong University, #247 Beiyuan Street, Jinan, 250033 China; 5 Department of Neurosurgery, First Affiliated Hospital of Wenzhou Medical University, Fanhai Xi Road, Wenzhou, 325000 China; 6 Department of Neurosurgery, Qilu hospital of Shandong University (Qingdao), #758 Hefei Road, Qingdao, 266035 China; 7 Department of Neurosurgery, Dezhou People’s Hospital, #1751 XinhuStreet, Dezhou, 253014 China

**Keywords:** CUL7, Glioma, NF-κB, MST1, miR-3940-5p

## Abstract

**Background:**

Cullin-7 (CUL7) is a member of the DOC domain-containing cullin family and is involved in the regulation of cell transformation. However, the clinical significance, potential mechanism and upstream regulators of CUL7 in malignant gliomas remain to be determined.

**Methods:**

Expression level data and clinical information were obtained via the Cancer Genome Atlas (TCGA) database, the Chinese Glioma Genome Atlas (CGGA) database, immunohistochemistry (IHC) and western blot analysis. Gene set enrichment analysis (GSEA) was used to explore the potential molecular mechanisms of CUL7. RNA silencing was performed using siRNA or lentiviral constructs in U87MG and U251 glioma cell lines and GSC267 glioma stem cells. CUL7 overexpression was performed using the GV141-CUL7 plasmid construct. In addition, overexpression of miR-3940-5p was performed and validated by quantitative real-time PCR (qRT-PCR). Cells were characterized in vitro or in vivo to evaluate their molecular status, cell proliferation, invasion, and migration by Cell Counting Kit (CCK)-8, EdU, flow cytometry, colony formation, Transwell and 3D tumour spheroid invasion assays. Coimmunoprecipitation (co-IP) and western blotting were performed to test the mechanisms of activation of the NF-κB signalling pathway.

**Results:**

High CUL7 expression was associated with a high tumour grade, a mesenchymal molecular glioma subtype and a poor prognosis in patients. Gene silencing of CUL7 in U87MG and U251 cells significantly inhibited tumour growth, invasion and migration in vitro and in vivo. Western blot analysis revealed that cyclin-dependent kinase inhibitors and epithelial-mesenchymal transition (EMT) molecular markers changed under CUL7 silencing conditions. In contrast, CUL7 overexpression promoted tumour growth, invasion and migration. Gene set enrichment analysis (GSEA) and western blot analysis revealed that CUL7 was positively associated with the NF-κB pathway. Moreover, with coimmunoprecipitation assays, we discovered that CUL7 physically associated with MST1, which further led to ubiquitin-mediated MST1 protein degradation, which promoted activation of the NF-κB signalling pathway. Finally, CUL7 was found to be downregulated by miR-3940-5p, which suppressed the development of gliomas.

**Conclusions:**

Our findings indicate that CUL7 plays a significant role in promoting tumorigenesis via NF-κB activation and that it can be negatively regulated by miR-3940-5p in human gliomas. Furthermore, CUL7 might be a candidate molecular target for the treatment of glioma.

## Background

Human glioma is the most common and fatal type of intracranial tumour, and it has an aggressive malignant progression represented by devastation to normal brain tissue, resistance to therapeutic approaches, and widespread invasion throughout the brain [1, 2]. The World Health Organization (WHO) classifies gliomas into four tumour grades (I–IV) according to their histopathology. Despite optimal treatment, patients with glioblastomas (GBM, WHO grade IV) still have a median survival of merely 12 to 15 months [3, 4].

In recent years, molecular signatures have become conspicuous in the classification of gliomas with improvements in gene technology. Based on gene expression signatures, glioma can be segregated into three subtypes (proneural, PN; classical, CL; and mesenchymal, MES) [5]. The mesenchymal subtype was identified as being particularly aggressive among these three subtypes [6–8]. Therefore, there is an urgent need to explore novel biomarkers and therapeutic targets for glioma that have been molecularly distinguished as the mesenchymal subtype.

Cullin-7 (CUL7), also known as KIAA0076, is a DOC domain-containing cullin assembling an SCF-ROC1-like E3 ubiquitin ligase complex including Skp1, CUL7, Fbx29, and ROC1 [9]. It has been reported that CUL7 is an oncogene directly involved in the regulation of cell transformation [10]. Furthermore, the upregulation of CUL7 has been revealed in multifarious malignant tumours, such as hepatocellular carcinoma [11], epithelial ovarian cancer [12], lung cancer [13], breast cancer [14], and choriocarcinoma [15]; thus, there is a good chance that CUL7 plays an important role in tumour progression. However, the expression level and clinical significance of CUL7 in human gliomas has not been confirmed. In addition, whether CUL7 is involved in the proliferation, apoptosis, invasion and migration of glioma remains unknown.

MST1 (mammalian sterile 20 like kinase 1) participates in the Hippo signalling pathway and was cloned by Chernoff’s group in 1995 [16]. Recently, it has been reported that MST1 inhibits TNFα-induced NF-κB signalling by regulating LUBAC activity [17]. MST1 plays a significant role in regulating gene expression, cell proliferation and apoptosis, and tumorigenesis [18].

MicroRNAs (miRNAs), a class of noncoding, small RNAs composed of 18–23 nucleotides, negatively regulate the expression of various genes at the posttranscriptional level by interacting directly with the 3′-untranslated regions (3′-UTRs) of their messenger RNAs (mRNAs) [19]. Many researchers have reported the involvement of miRNAs in the diversity of carcinogenesis. Dysregulation of miRNAs has been recognized to be associated with a variety of disorders, particularly cancers [20]. However, there is still inadequate understanding of miRNA interactions with vital signalling pathways in gliomas.

In this study, we demonstrated that CUL7 acted as a novel oncogene associated with NF-κB activation in glioma, and we investigated its relationship with miR-3940-5p, a microRNA that has been reported to inhibit cell proliferation [21]. Our results revealed that overexpression of miR-3940-5p could reduce the expression of CUL7 and inhibit proliferation, invasion and migration in gliomas.

## Materials and methods

### Clinical specimens and databases

The data for the mRNA expression microarrays and the attendant clinical information for the samples were downloaded from The Cancer Genome Atlas Research Network (*n* = 603; TCGA, http://cancergenome.nih.gov) and were used for the analysis. In addition, the Chinese Glioma Genome Atlas (*n* = 301; CGGA, http://www.cgga.org.cn), an external independent glioma database, was also mined. Archived paraffin embedded glioma tissues (WHO grades I–IV) were gathered from patients (*n* = 38) who underwent surgery in the Department of Neurosurgery, Qilu Hospital of Shandong University. Normal brain tissue samples (*n* = 4) were collected from severe traumatic brain injury patients who experienced partial resection of the normal brain as decompression treatment.

### Immunohistochemistry (IHC)

Sections were obtained from formalin-fixed, paraffin-embedded tissues of different grades of human gliomas and normal brains. Sections were heated, deparaffinized, rehydrated and placed in sodium citrate buffer (pH 6.0) for antigen retrieval, and endogenous HRP activity was blocked with 3% hydrogen peroxide (H_2_O_2_). The slides were blocked with 10% normal goat serum and incubated with primary antibodies (mouse anti-CUL7 antibody, Santa Cruz, USA; rabbit anti-Ki67 antibody, Cell Signaling Technology, USA) at 4 °C overnight. The signal was visualized using standard protocols with horseradish-peroxidase-conjugated secondary antibodies and 3, 3′-diaminobenzidine (DAB) as the substrate. The sections were incubated with normal mouse serum as negative controls. Then, the slides were counterstained with haematoxylin, and typical images were obtained using a Leica DM 2500 microscope. The protein expression were scored by Image Pro Plusversion (IPP) image processing software.

### Gene set enrichment analysis (GSEA)

To gain insight into the biological processes and signalling pathways associated with CUL7 expression in gliomas, GSEA was performed using the Broad Institute GSEA version 4.0 software. The TCGA database was downloaded. The gene sets used for the enrichment analysis were downloaded from the Molecular Signatures Database (MsigDB, http://software.broadinstitute.org/gsea/index.jsp).

### Cell culture

U87MG, U251 and A172 human glioma cell lines and normal human astrocytes (NHA) were obtained from the Culture Collection of the Chinese Academy of Sciences (Shanghai, China) and cultured in Dulbecco’s modified Eagle’s medium (DMEM; Thermo Fisher Scientific; USA) that was supplemented with 10% foetal bovine serum (FBS; Gibco; USA). GSC 267 were kindly provided by Dr. Krishna P.L. Bhat (The University of Texas, M.D. Anderson Cancer Center, Houston, TX) and cultured in DMEM/F12 media supplemented with B27 (Invitrogen, USA), EGF (R&D Systems, USA), and bFGF (R&D Systems, USA). These cells were maintained at 37 °C in a humidified chamber containing 5% CO2.

### CUL7 silencing and overexpression

Small interfering RNA (siRNA) targeting CUL7 were synthesized (GenePharma; Shanghai, China). siRNAs were transfected with Lipofectamine™ 3000 reagent (Thermo Fisher Scientific; USA) according to the manufacturer’s protocol. Stable knockdown of CUL7 in cells was generated using lentiviral transduction of shCUL7 (Genepharma; Shanghai, China). Knockdown efficiency was assessed 48 h after transfection by western blotting. Negative control (NC) sequences are as follows: 5′-UUCUCCGAACGUGUCACGUTT-3′; siRNA sequences that generated efficient knockdown are as follows: si-CUL7#1: 5′-UGAGAUCCUAGCUGAACUGTT-3′; and si-CUL7#2: 5′-AGAACUCCGCUACAGGGAAUU − 3′. Plasmid construction of GV141-CUL7 was performed by GeneChem (Shanghai, China). Cells were transfected with GV141-CUL7 to induce the overexpression of CUL7 and with empty GV141 vector (GV141) as a control.

### Quantitative real-time PCR (qRT-PCR)

Total RNA was isolated from glioma cells using TRIzol reagent (Invitrogen, Life Technologies). Reverse transcription was performed using High Capacity cDNA Reverse Transcription Kits (Applied Bio-systems) according to the manufacturer’s protocols. The cDNA was subjected to real-time PCR using the quantitative PCR System Mx-3000P (Stratagene). The sequences of the PCR primers are as follows: hsa-miR-3940-5p F primer, 5′-TAAAAGTGGGTTGGGGCGG-3′, R primer, 5′-GTGCAGGGTCCGAGGT-3′; U6 F primer, 5′-CAGCACATATACTAAAATTGGAACG-3′, R primer, 5′-ACGAATTTGCGTGTCATCC-3′; CUL7 F primer, 5′ -ACCTGAAGGCGGTCTCTGT-3′, R primer, 5′-CCTTGCTGCCATCTCGAATC-3′; MST1 F primer, 5′-GGCCTTCCACTACAACGTGA-3′, R primer, 5′-GCAGGTCCGTACGTAGTCTTT-3′; GADPH F primer, 5′-GCACCGTCAAGGCTGAGAAC-3′, R primer: 5′-TGGTGAAGACGCCAGTGGA-3′. The relative miRNA/mRNA expression normalized to U6/GAPDH was analysed using GraphPad Prism 6 software.

### Western blotting

Harvested cells were lysed with heat denaturation in RIPA cell lysis buffer. Protein lysates (20 μg) were loaded and separated on SDS-PAGE, and the proteins were transferred to polyvinylidene difluoride (PVDF) membranes. The blots were incubated with primary antibodies against CUL7, cleaved-PARP, Ub (Santa Cruz, USA); p21, p27, Cyclin D1, CDK4, Cyclin E1, CDK2, MST1, Phospho-IKK, IKKα, Phospho-IκBα, IκBα, Phospho-NF-κB p65, NF-κB p65, Bcl-2, Bax, cleaved-caspase3, N-cadherin, Vimentin, Slug, Twist, MMP2, GAPDH (Cell Signaling Technology, USA); and E-cadherin, MMP9 (Proteintech, China). Specific proteins were visualized with enhanced chemiluminescence (ECL, Millipore, Bredford, USA). The intensity of the protein bands was measured (ImageJ software) and normalized to GAPDH.

### Co-immunoprecipitation

Cells were lysed in IP buffer (Pierce, Rockford, USA) including protease inhibitor cocktail (Sigma). The lysates were incubated with 5 μg appropriate primary antibodies or IgG and Protein A/G agarose beads (Pierce) overnight at 4 °C with gentle shaking. The immunoprecipitated complexes were then washed with lysis buffer three or four times and eluted from the beads with protein loading buffer. Western blotting analysis was then performed for detection of proteins.

### Cycloheximide (CHX) chase

Cells were infected with siRNA targeting CUL7. After 48 h, CHX (25 μg/mL) was added to the culture medium to inhibit translation, and cells were incubated for 0, 2, 4, or 6 h. Cell lysates were prepared, and protein was examined using western blot analysis. Experiments were performed in triplicate.

### Cell counting kit (CCK)-8 assay

The Cell Counting Kit-8 (CCK-8) was used to evaluate the cell viability according to the manufacturer’s instructions (Dojindo, Japan). U87MG or U251 cells (5 × 10 ^3^ cells/well) were incubated in 96-well plates for 24, 48, and 72 h. The CCK-8 solution (10 μL) was added to each well and the plates were incubated for 1 h at 37 °C, and then the absorbance at 450 nm wavelength (OD450) was measured in a Microplate Reader (Bio-Rad).

### 5-ethynyl-2′-deoxyuridine (EdU) cell proliferation assay

Cell proliferation rates were measured by an EdU cell proliferation assay kit (RiboBio, #C10310–1; China). Cells were incubated with 200 μL of 5-ethynyl-20-deoxyuridine (EdU) for 2 h at 37 °C. Cells were fixed in 4% paraformaldehyde for 20 min, permeabilized with 0.4% Triton X-100 for 10 min, and incubated with Apollo® reagent (100 μL) for 30 min. The cells were stained with Hoechst for 30 mins, and representative images were obtained using a Nikon inverted fluorescence microscope. The cell proliferation rate was assessed using the ratio of EdU positive cells (Red) to the total Hoechst positive cells (Blue).

### Flow cytometry

Cell cycle analysis was performed by determining the DNA content with propidium iodide (PI) staining (BD Biosciences; San Jose, CA, USA). Briefly, U87MG and U251 glioma cells were harvested, re-suspended and stained with propidium iodide (PI; BD Biosciences) in the presence of RNase A for 20 min. Apoptosis was evaluated in the U87MG and U251 cells with Annexin V-FITC and PI staining (15 min; BD Biosciences). Cells were analysed using a flow cytometer (BD Biosciences) according to the manufacturer’s instructions.

### Colony formation assay

For the colony formation assay, cells were seeded in 6-well plates at a density of 2000 cells/well. The DMEM containing 10% FBS was changed every third day. After 15 days, the colonies were fixed with 4% paraformaldehyde for 30 mins and stained with crystal violet for 15 mins, and representative colonies were imaged and quantified.

### 3D tumour spheroid invasion assay

Glioma spheroids were generated by incubating cells in the spheroid formation matrix for 72 h in a 3D culture qualified 96-well spheroid formation plate. Spheroids with a diameter of > 200 mm were embedded into the invasion matrix (Trevigen, Gaithersburg, USA) composed of basement membrane proteins in the 96-well plate. Glioma spheroids were photographed every 24 h under Nikon microscopy. The spheroids at 0 h were used as a reference point for measurement of the area invaded by sprouting cells.

### Transwell invasion and migration assays

To further assess invasiveness, the filters were pre-coated with Matrigel. Glioma cells were added to the top chamber in serum-free media. The bottom chamber was filled with 10% FBS DMEM. After 24–48 h of incubation, the top chamber cells were removed using a cotton swab, and the membrane was fixed in 4% paraformaldehyde for 15 min and stained with crystal violet for 15 min. Five fields of adherent cells in each well were photographed randomly. To measure migration, the filters were not pre-coated with Matrigel.

### Tumorsphere formation assay

GSCs were plated in 48-well plates at a density of 2000 cells per well with 250 μL of the GSC culture media mentioned above, and the transfections were performed. Three to four days later, an additional 70 μL of the aforementioned treatment was added. Tumorsphere numbers were calculated on the seventh day after cell placement.

### Intracranial mouse model

To establish intracranial gliomas, U87MG luciferase cells (3 × 10^5^) were transfected with Lenti-sh-CUL7 or Lenti-Control virus and then stereotactically implanted into the brains of 4-week-old nude mice (SLAC Laboratory Animal Center; Shanghai, China). Bioluminescence imaging was used to detect intracranial tumour growth on days 5, 10, 15, 20 and 25. Kaplan-Meier survival curves were plotted to determine the survival time and weight. Tumour tissues were harvested at 15 days after implantation, fixed in formalin, embedded in paraffin, sectioned and incubated with antibodies against CUL7 (Santa Cruz, USA), MST1(Abcam, UK), Ki-67 (Cell Signaling Technology, USA) and N-cadherin (Cell Signaling Technology, USA).

### Luciferase reporter assays

HEK293 cells were cotransfected with firefly luciferase reporters and the indicated plasmids using Lipofectamine 3000 (Invitrogen/Thermo Fisher Scientific), and luciferase assays were performed 24 h later using the Dual Luciferase Reporter Assay Kit (Promega). Renilla activity was used to normalize the luciferase reporter activity. The reporter genes containing GV272-CUL7 and GV272-mutCUL7 were synthesized by Genechem (Shanghai, China).

### Statistical analysis

Survival curves were estimated by the Kaplan-Meier method and compared using the log-rank test. The cut-off level was set at the median value of the CUL7 expression levels. The expression pattern of CUL7 in different glioma subtypes and the associations of CUL7 with isocitrate dehydrogenase 1 (IDH1) mutations, methylation of the O-methylguanine-DNA methyltransferase (MGMT) promoter, codeletion of 1p/19q, telomerase reverse transcriptase (TERT) loss, and alpha thalassemia/ mental retardation syndrome X-linked (ATRX) mutation were performed using the TCGA dataset. A two-tailed χ2 test was used to determine the association between CUL7 expression and clinicopathological characteristics. Pearson correlation was used to evaluate the linear relationship between the expression of different genes. A one-way ANOVA test or Student’s t test were used for all other data comparisons using the Statistical Product and Service Solutions (SPSS) software. All data are presented as the mean ± standard error. All tests were two-sided, and *P*-values < 0.05 were considered to be statistically significant. The experimental graphs were generated using GraphPad Prism 6 software.

## Results

### CUL7 is upregulated in high-grade and mesenchymal subtype glioma patients and indicates a poor prognosis

**Fig. 1:**
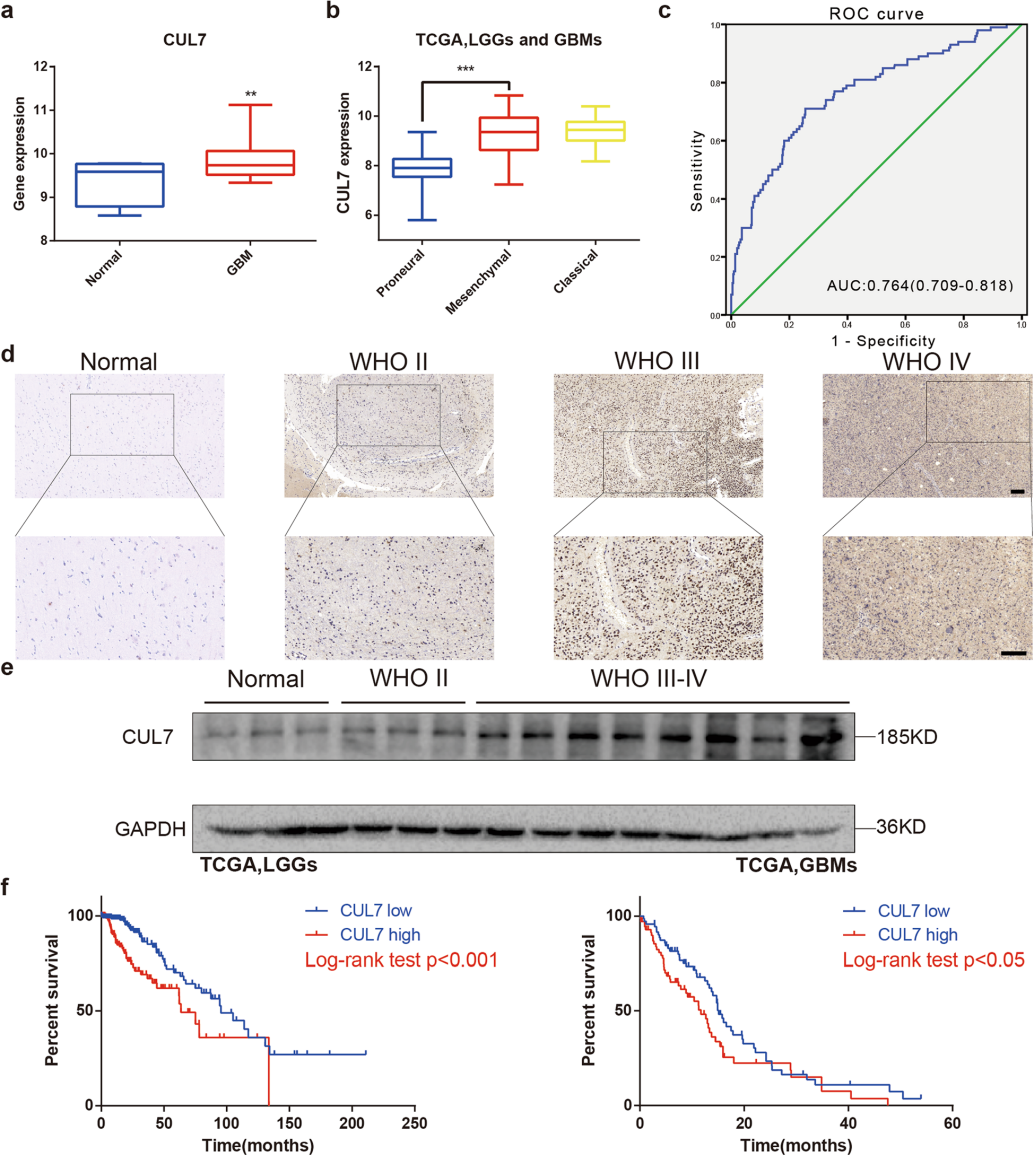
Expression of CUL7 is associated with tumor grade and patient survival in gliomas**. a** Quantification of CUL7 mRNA expression levels in GBMs and normal brain tissues in TCGA. **b** Quantification of gliomas subtype-specific CUL7 expression in TCGA. Log2-transformed expression of CUL7 mRNA levels are listed on the Y-axis. Error bars represents the SEM. **c** ROC curve showing sensitivity of CUL7 as a marker to discriminate between mesenchymal subtype and non-mesenchymal subtype glioma patients. **d** Representative images of IHC staining for CUL7 in different grade gliomas and normal brain tissues (scale bar = 100 μm). **e** Western blot analysis of CUL7 levels in lysates from different grades of glioma tissues (WHO grades II–IV) and normal brain tissues. **f** Kaplan–Meier survival analysis for glioma patients with high CUL7 expression and low CUL7 expression in LGGs (*n* = 457) or GBMs (*n* = 141) in TCGA database. The cut-off level was set at the median value of the CUL7 levels. *P < 0.05; **P < 0.01; ***P < 0.001

To understand the function of CUL7 in glioma development, we first analysed the mRNA levels of CUL7 in glioblastoma (GBM) tissues and normal brain tissues from the TCGA dataset. Compared to normal brain tissues, the gene expression levels of CUL7 were significantly increased in GBM tissues (Fig. 1a). We also validated these findings in the CGGA dataset (Fig. S1a).

Gliomas have been classified into three distinct molecular subtypes: proneural, mesenchymal and classical. Compared to the proneural subtypes, the mesenchymal subtype has been associated with a poor prognosis in patients [22]. Therefore, we examined the mRNA level of CUL7 in the TCGA and CGGA on the basis of the molecular subtype. The expression level of CUL7 was significantly higher (> median value) in the mesenchymal subtype than in the proneural subtype (Fig. 1b; Fig. S1b; Table 1), and the ROC curve further showed the sensitivity of CUL7 as a marker to discriminate between the mesenchymal subtype and the non-mesenchymal subtype glioma patients (Fig. 1c). Moreover, the protein levels of CUL7 were examined by immunohistochemical staining in an unconnected cohort of glioma and normal brain tissue samples from Qilu Hospital. Correspondingly, the expression of the CUL7 protein was higher in high-grade gliomas (WHO III-IV; *n* = 21) than in normal brain tissues (*n* = 4) and low-grade gliomas (WHO I-II; *n* = 17; Fig. 1d; Fig. S1c; Table 2). These findings were further confirmed by western blot analysis (WHO II-IV; *n* = 11; normal brain tissues; *n* = 3; Fig. 1e; Fig. S1d). Thus, high CUL7 expression was correlated with an increased tumour grade in glioma patients.

**Table 1:** Correlation of CUL7 expression in human glioma patients with different clinicopathological features. *P* values were determined by the Chi-square and Fisher’s exact tests

Variable		CUL7 high expression	CUL7 low expression	*p* value
Age	≥45	218	114	< 0.001
	< 45	95	190	
Gender	Male	183	178	0.983
	Female	130	126	
KPS	≥80	161	154	0.030
	< 80	44	23	
WHO grade	II	71	145	< 0.001
	III	106	135	
	IV	136	24	
TCGA subtype	Neural	77	36	< 0.001
	Proneural	53	186	
	Classical	85	2	
	Mesenchymal	81	19	
IDH status	Mutant	220	317	< 0.001
	Wild-type	113	18	
MGMT promotor	Methylated	179	299	< 0.001
	Unmethylated	129	36	
1p/19q	Codeletion	41	128	< 0.001
	Non-codeletion	294	208	
TERT expression	Not expressed	121	195	< 0.001
	Expressed	209	141	
ATRX status	Mutant	54	143	< 0.001
	Wild-type	273	192

**Table 2:** Association between CUL7 expression and clinicopathologic factors in glioma

Variable		CUL7 high expression	CUL7 low expression	*p* value
Age	≥45	13	7	0.022
	< 45	5	13	
Gender	Male	10	11	0.973
	Female	8	9	
WHO grade	I-II	3	14	< 0.001
	III-IV	15	6	
Ki67 positive %	≥15	11	3	0.003
	< 15	6	16	
Volume (cm^3^)	≥35	7	8	0.653
	< 35	7	11	

The expression levels of CUL7 were also associated with the clinicopathological characteristics of patients in the TCGA. Age (≥ 45 years) and KPS (< 80; Table 1) were statistically associated with high CUL7 expression when comparing the median values. In addition, we analysed whether CUL7 expression was correlated with some molecular genetic characteristics, including IDH1 mutations, MGMT promotor methylation, 1p/19q codeletion, low TERT expression level, and ATRX mutations, because these factors have been found to be associated with a beneficial prognosis in gliomas [23, 24]. A significantly higher number of patients with low CUL7 expression levels possessed these advantageous characteristics.

We also examined the prognostic value of CUL7 using data from LGG (*n* = 457) and GBM (*n* = 141) patients in the TCGA. Kaplan-Meier survival analysis revealed that participants with high expression levels of CUL7 had a shorter overall survival (OS) than those with low expression levels of CUL7 (63.51 vs 95.51 months, *p* < 0.001 in LGG, 11.73 vs 15.11 months, *p* < 0.05 in GBM) (Fig. 1f). These results were validated in CGGA (*n* = 285) (Fig. S1c). Furthermore, CUL7 expression was confirmed as an independent indicator of OS in glioma after multivariate Cox regression analysis (HR = 3.084, 95% CI = 1.918 to 4.959, *P* < 0.0001; Table 3). Therefore, CUL7 might be a novel prognostic biomarker in gliomas.

**Table 3:** Univariate and multivariate Cox regression of CUL7 expression for overall survival in glioma patients

Variable	Univariate Cox Regression		Multivariate Cox Regression	
	HR (95% CI)	p	HR (95% CI)	p
Age	4.006 (3.208–5.004)	< 0.001	2.549 (1.673–3.883)	< 0.001
Old vs young				
Gender	1.106 (0.926–1.321)	0.27	0.952 (0.700–1.295)	0.754
male vs female				
WHO grade	7.175 (5.606–9.182)	< 0.001	3.004 (1.926–4.683)	< 0.001
High- vs low-				
TCGA subtype	2.300 (1.882–2.810)	< 0.001	1.582 (1.099–2.278)	0.014
Mesenchymal vs non-mesenchymal				
CUL7 expression	6.582 (4.578–9.465)	< 0.001	3.084 (1.918–4.959)	< 0.001
High vs low				

### Analysis of the potential biological functions of CUL7 by GSEA

GSEA based on CUL7 expression in the TCGA database was performed to explore the potential biological functions of CUL7 in gliomas. Interestingly, high expression of CUL7 was associated with cell proliferation, apoptosis and epithelial-mesenchymal transition (EMT) (Fig. S2a-d). GSEA results further confirmed that CUL7 positively correlated with the Phillips-queried MES gene set but negatively correlated with the PN gene set (Fig. S2e) [25]. Therefore, CUL7 is implicated in the malignant properties of CUL7 in gliomagenesis.

### CUL7 silencing results in cell cycle arrest and apoptosis in glioma cells in vitro

**Fig. 2:**
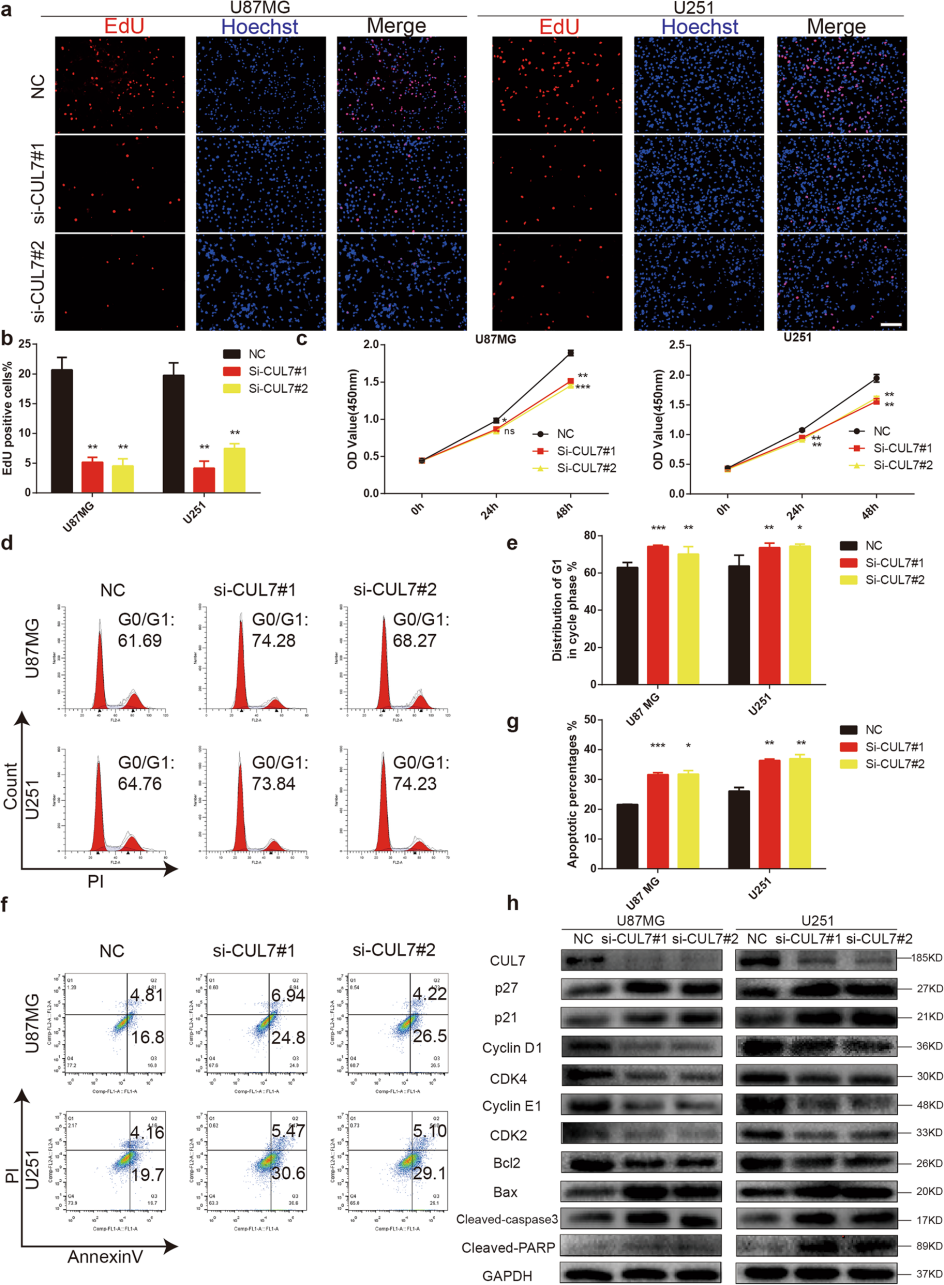
CUL7 knockdown inhibits cell proliferation and induces cell cycle arrest and cell apoptosis**.** U87MG and U251 cells transfected with CUL7 siRNA or controls and characterized in the following assays: (**a, b**) EdU performed 48 h after transfection (scale bar = 100 μm); (**c**) growth curve based on OD450 using the CCK-8 assay; (**d, e**) cell cycle profiles determined from PI staining in flow cytometry; (**f, g**) % apoptosis as determined with Annexin V-FITC antibody and PI staining in flow cytometry. (**h**) Western blot to detect expression levels of the known cell cycle and apoptosis regulatory factors indicated. GAPDH was used as a loading control. Data are represented as the mean ± SEM from three independent experiments. *P < 0.05; **P < 0.01; ***P < 0.001, relative to control.NC: non-silencing siRNA; si-CUL7: siRNAs targeting CUL7

Next, a series of experiments were performed to assess the role of CUL7 in several cellular processes of human glioma cells. We designed two independent siRNA sequences against CUL7. Both demonstrated efficient silencing of CUL7 in U87MG and U251 cells, two malignant glioma cell lines (Fig. 2h). To directly assess the role of CUL7 in glioma cell survival and proliferation, we performed EdU and CCK-8 assays in cells that were or were not transfected with siRNA. Downregulation of CUL7 resulted in a statistically significant decrease in the percentage of EdU-positive cells as well as changes in OD450 values in both U87MG and U251 cells 48 h after transfection (Fig. 2a-c). In addition, these findings were further confirmed by colony forming assays (Fig. S3a-b). Cell cycle analysis by flow cytometry also demonstrated that silencing CUL7 with a single siRNA increased the proportion of U87MG and U251 cells in the G0/G1 phase (Fig. 2d-e). Furthermore, knockdown of CUL7 increased the percentage of apoptotic cells in the U87MG and U251 cell lines (Fig. 2f-g). Moreover, the prominent effect of CUL7 silencing on the self-renewal abilities of GSCs was verified by a tumoursphere formation assay (Fig. S3c-d).

Next, we used western blotting to thoroughly investigate the downstream targets of CUL7 that influenced the cell cycle and apoptosis. A previous study reported that CUL7 could degrade tumour suppressors, including p21 and p27 [13, 26]. In our study, p21 and p27, which were identified as cyclin-dependent kinase inhibitors [27, 28], were present in higher amounts in the si-CUL7 group in gliomas than in the controls (Fig. 2h). In contrast, the expression of cyclin-dependent kinase 2 (CDK2)/cyclin E1 and cyclin-dependent kinase 4 (CDK4)/cyclin D1 was decreased after CUL7 silencing (Fig. 2h). Taken together, these results suggest that loss of CUL7 suppressed cell cycle progression and caused apoptosis in human glioma cells.

### Knockdown of CUL7 reduces invasion and migration in human glioma cells

**Fig. 3:**
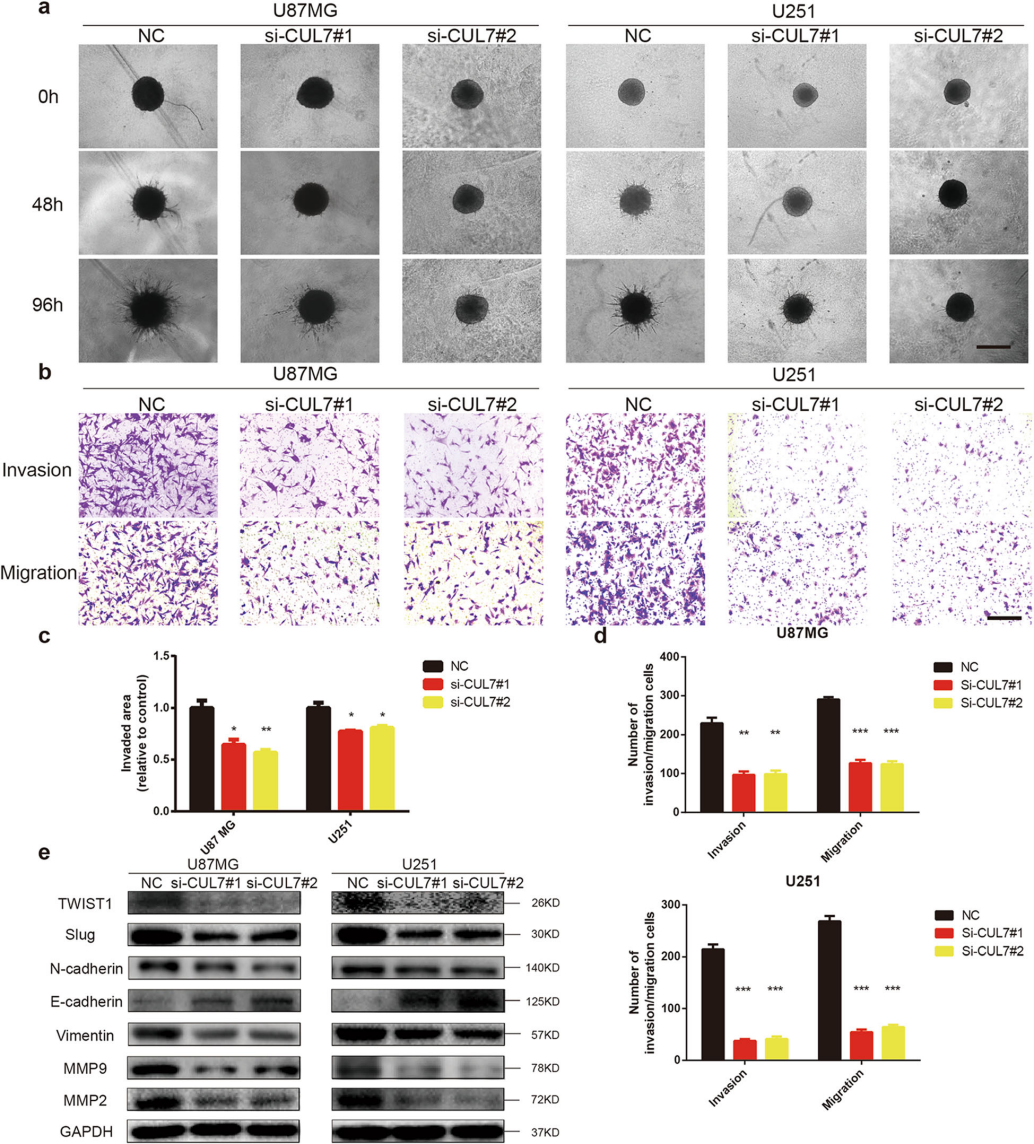
CUL7 knockdown decreases invasive and migrative ability of glioma cells**. a** Representative images of invaded spheroids in 3D invasion assay for U87MG and U251 transfected with CUL7 and control siRNAs evaluated at 48 h and 96 h are shown (Scale bar = 200 μm). **b** Representative images of Transwell migration and invasion assays performed in U87MG and U251 transfected NC and si–CUL7 cells (Scale bar = 200 μm). **c** The area covered by invading cells quantitated after 96 h. **d** Graphic representation of migrated and invaded cells counts from Transwell assay. Data are represented as the mean ± SEM from three independent experiments. *P < 0.05; **P < 0.01; ***P < 0.001, relative to control. **e** Western blot for protein levels of EMT components in lysates (20 μg) from U87MG and U251 cells transfected with siRNA against CUL7 and controls. GAPDH was used as a loading control. NC: non-silencing siRNA; si-CUL7: siRNAs targeting CUL7

To assess the role of CUL7 in cell invasion and migration, 3D collagen spheroid invasion assays and Transwell chamber assays were performed. In the 3D collagen spheroid invasion assay, the area invaded by the U87MG and U251 spheroids in the CUL7 knockdown groups was reduced relative to that in the controls (Fig. 3a, c). Similarly, the results of the Transwell invasion assays also revealed that CUL7 silencing reduced the number of glioma cells invading the Matrigel-coated membrane compared with that in the control groups (Fig. 3b, d). Moreover, the same results were found with the Transwell migration assay (Fig. 3b, d).

In addition to GSEA analysis, a number of reports have demonstrated that CUL7 induces epithelial-mesenchymal transition (EMT) [15, 29], which plays a significant role in the invasiveness and metastasis of several cancers [30–32]. The results of western blotting revealed that knockdown of CUL7 inhibited EMT by decreasing several mesenchymal markers, such as TWIST1, N-cadherin, Vimentin and Slug, and increased the expression of epithelial factors (E-cadherin) (Fig. 3e). Furthermore, CUL7 silencing induced the downregulation of the expression of MMP2 and MMP9, matrix metalloproteinase (MMP) family proteins that play important roles in ECM degradation and in the migration and invasion of tumour cells [33] (Fig. 3e). These results demonstrated that CUL7 promoted the migration and invasion of human glioma cells.

### Overexpression of CUL7 promotes proliferation, migration and invasion in glioma cells

**Fig. 4:**
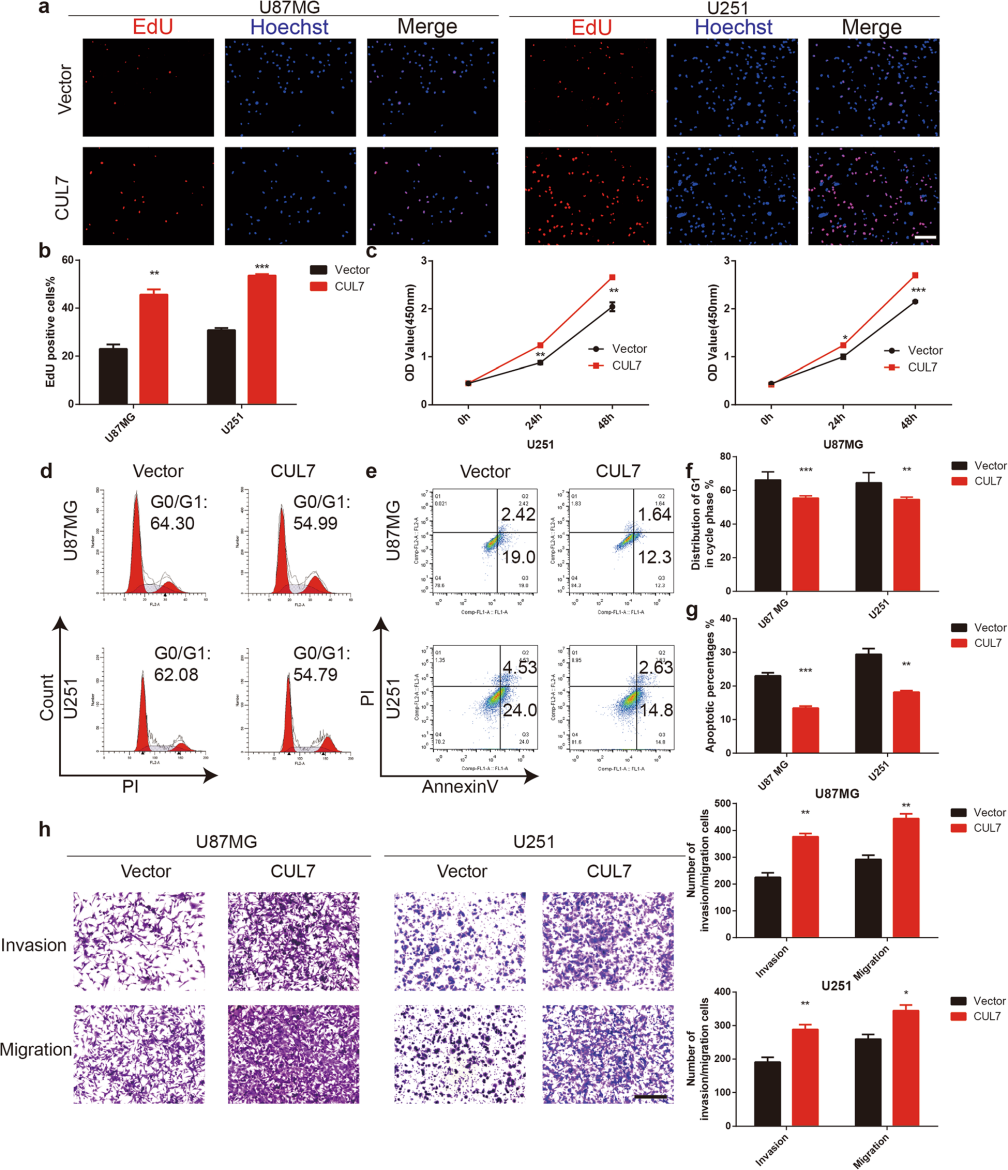
CUL7 overexpression promotes proliferation, invasion and migration of glioma cells**.** U87MG and U251 cells transfected with GV141-CUL7s or Vectors and characterized in the following assays: (**a, b**) EdU performed 48 h after transfection (scale bar = 100 μm); (**c**) growth curve based on OD450 using the CCK-8 assay; (**d, f**) cell cycle profiles determined from PI staining in flow cytometry (**e, g**) % apoptosis as determined with Annexin V-FITC antibody and PI staining in flow cytometry. **h** Representative images of Transwell migration and invasion assays performed in U87MG and U251 cells. Graphic representation of migrated and invaded cells counts from Transwell assay. Data are represented as the mean ± SEM from three independent experiments (Scale bar = 200 μm). *P < 0.05; **P < 0.01; ***P < 0.001, relative to control. Vector: GV141-empty; CUL7: GV141-CUL7

To further study the effect of CUL7 overexpression on the proliferation, invasion and migration of glioma cells, cells were transfected with the GV141-CUL7 plasmid to overexpress CUL7. EdU and CCK-8 assays were performed. Upregulation of CUL7 resulted in an increase in the percentage of EdU-positive cells and the OD450 values in both U87MG and U251 cells (Fig. 4a-c). Cell cycle analysis also demonstrated that overexpression of CUL7 decreased the population of U87MG and U251 cells in G0/G1 phase (Fig. 4d, f). Furthermore, overexpression of CUL7 decreased the percentage of apoptotic cells in the U87MG and U251 cell lines (Fig. 4e, g). In the Transwell assays, the number of migrated or invaded glioma cells in the CUL7 overexpression groups was increased compared with that in the control groups (Fig. 4h). Moreover, western blot analysis verified that the protein expression levels of cyclin E1, CDK2, N-cadherin, Slug, MMP2 and MMP9 increased while those of p27, p21 and E-cadherin decreased in the CUL7 overexpression group (Fig. S3e).

### CUL7 promotes gliomagenesis by activating the NF-κB pathway

**Fig. 5:**
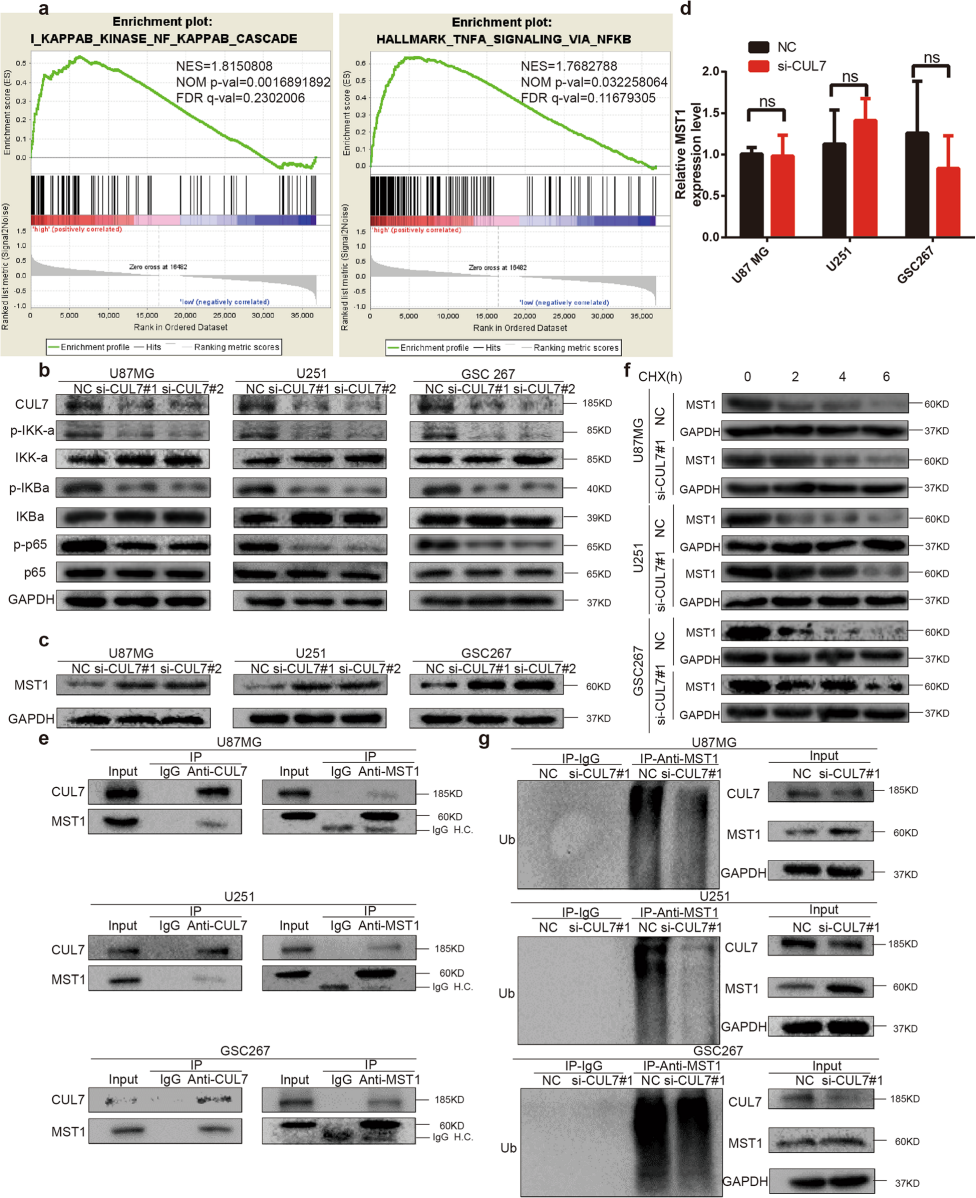
CUL7 leads to ubiquitin-mediated MST1 protein degradation and promotes activation of the NF-κB pathway. **a** GSEA highlighting positive association of increased CUL7 expression levels with NF-κB signal pathway. NES = normalized enrichment score; NOM = nominal FDR = false discovery rate. **b** Western blot for protein levels of NF-κB pathway components in lysates (20 μg) from U87MG, U251 and GSC267 cells transfected with siRNA against CUL7 and controls. GAPDH was used as a loading control. **c** Western blot for protein levels of MST1 in lysates (20 μg) from U87MG, U251 and GSC267 cells transfected with siRNA against CUL7 and controls. **d** qRT-PCR analysis of MST1 in U87MG, U251 and GSC267 cells. Expression is normalized to GAPDH mRNA. Data are represented as the mean ± SEM. **e** Western blot analysis of co-precipitating proteins in IPs performed using anti-CUL7 or -MST1 antibody on lysates prepared from U87MG, U251 and GSC267-NC, si-CUL7 cells. **f** Western blot analysis of MST1 protein in modified U87MG, U251 and GSC267 cells treated with CHX (25 μg/mL) for the indicated time. **g** Western blot analysis of MST1 IPs performed on lysates prepared from U87MG, U251 and GSC267 cells treated with MG132 (20 μM) for 8 h to examine endogenous MST1 ubiquitination. NC: non-silencing siRNA; si-CUL7: siRNAs targeting CUL7

To investigate the regulatory mechanism of CUL7 in glioma progression, we used GSEA to explore the potential pathways of CUL7 and found that CUL7 was positively associated with the NF-κB pathway (Fig. 5a). Interestingly, gene silencing of CUL7 in U87MG, U251 and GSC267 cells significantly inhibited the phosphorylation of IKK-α and IκBα, the phosphorylation of NF-κB (p65), and the translocation of p65 to the nucleus, inhibiting the activity of the NF-κB pathway (Fig. 5b). In contrast, overexpression of CUL7 increased the activity of the NF-κB pathway. (Fig. S3f). These results demonstrated that CUL7 promoted the activation of the NF-κB pathway.

### CUL7 promotes MST1 protein ubiquitous degradation and activation of the NF-κB pathway

In 2018, Zou et al. found that CUL7 mediates the ubiquitination and degradation of MST1 [34]. Mammalian sterile 20-like 1 (MST1) functions as a suppressor in glioma [35, 36]. In addition, we detected MST1 protein levels in glioma tissues of different grades by IHC. Interestingly, the results demonstrated that MST1 protein levels were downregulated in high-grade glioma tissues (Fig. S3h). In addition, we determined if MST1 expression was correlated with expression of CD44, a representative mesenchymal marker [25, 37, 38]. As shown in Fig. S3h, expression of MST1 and CD44 were negatively correlated, which demonstrated that MST1 protein levels were downregulated in mesenchymal subtype glioma tissues. Lee et al. recently established that MST1 negatively regulates the NF-κB pathway [17]. In our study, MST1 protein levels were higher in the si-CUL7 group than in the control group (Fig. 5c). In contrast, overexpression of CUL7 decreased the protein level of MST1 (Fig. S3f). To determine the mechanism by which CUL7 promotes the degradation of MST1, we examined whether CUL7 regulates MST1 transcriptionally. However, MST1 mRNA levels were not significantly different in the si-CUL7 groups compared with the control groups (Fig. 5d). Therefore, we tested whether CUL7 modulates the degradation of MST protein. To do this, we performed a series of immunoprecipitations (IPs) to pulldown complexes by using antibodies against CUL7 or MST1. Both CUL7 and MST1 were found in complexes with antibodies against CUL7 or MST1 (Fig. 5e). In addition, the half-life of MST1 protein was altered in the si-CUL7 group. In the presence of the protein synthesis inhibitor cycloheximide (CHX), we found that the half-life of MST1 was prolonged in U87MG-, U251- and GSC267-si-CUL7 cells (Fig. 5f; Fig. S2f). Finally, endogenous MST1 ubiquitination was decreased in the si-CUL7 group (Fig. 5g). Therefore, we found that CUL7 physically associated with MST1 and furthermore led to ubiquitin-mediated MST1 protein degradation, which promoted activation of the NF-κB signalling pathway [17].

### Downregulation of CUL7 inhibits glioma tumorigenesis in vivo

**Fig. 6:**
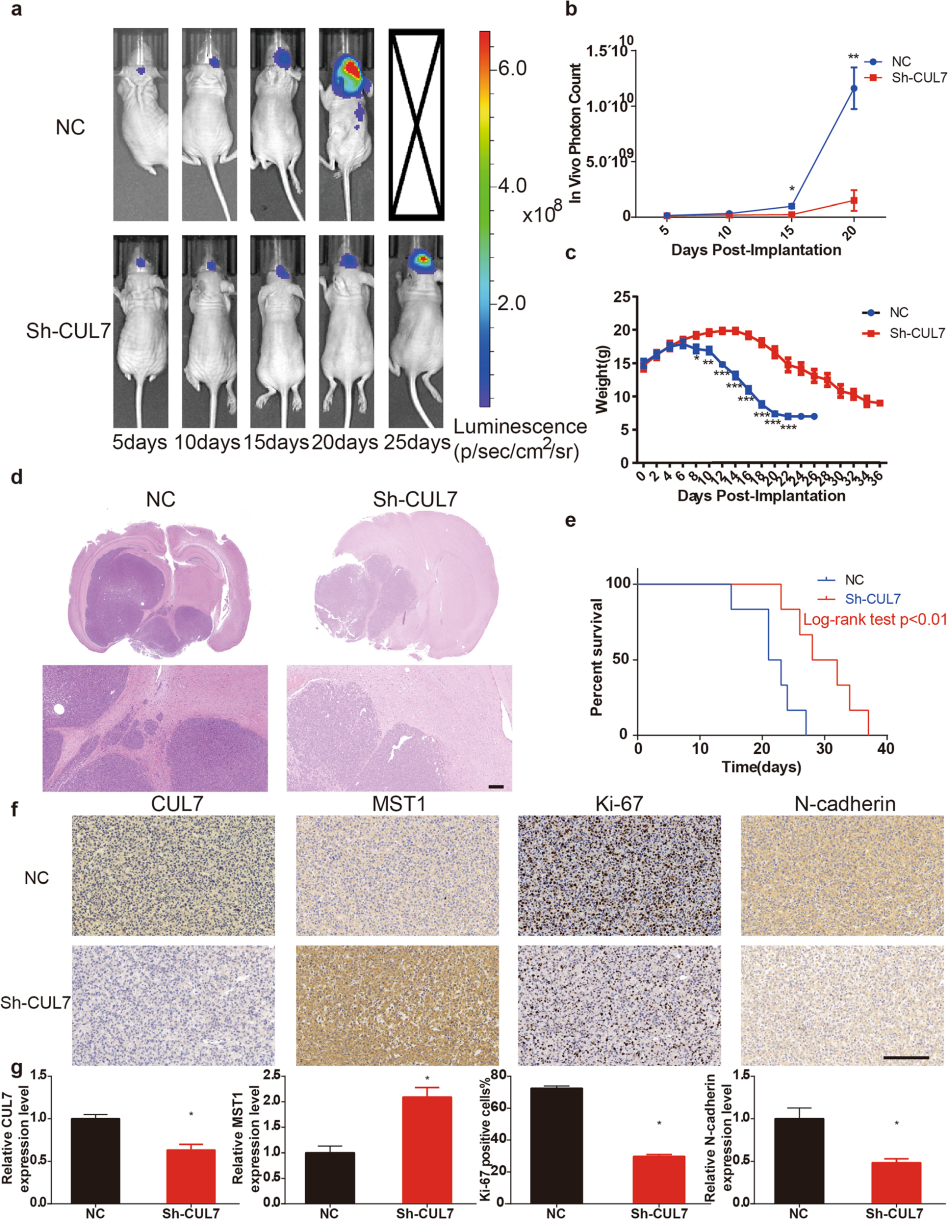
CUL7 silencing inhibits tumorigenesis in vivo. **a, b** Bioluminescence imaging showed the tumor size as days elapsed. **c** Weights of two groups of mice were measured every two days. **d** H&E staining of sections from mouse brains with U87MG control or sh-CUL7 xenografts at 15 days after implantation with 3 × 106 cells. (scale bar = 200 μm) **e** Survival analysis for animals implanted with U87MG sh-CUL7 or control cells (P < 0.01 by log-rank test; n control = 6, n sh-CUL7 = 6). **f, g** IHC for CUL7, MST1, Ki-67 and N-cadherin in sections from indicated xenografts (scale bar = 100 μm). Data are represented as the mean ± SEM

Furthermore, modified U87MG luciferase cells were implanted in nude mice orthotopically to verify the function of CUL7 in gliomas in vivo. The expression level of CUL7 was downregulated in U87MG luciferase cells transfected with Lenti-sh-CUL7 (Fig. S3g). The tumour sizes examined by bioluminescence imaging were reduced in animals bearing sh-CUL7 cells (Fig. 6a-b), and the weight loss of the sh-CUL7 group was much slower than that of the control group (Fig. 6c). HE staining of tumours collected 15 days after implantation in the two groups displayed a smaller size and more defined borders in the sh-CUL7 group (Fig. 6d). Moreover, the survival rate of animals implanted with U87MG-sh-CUL7 was longer than that of the controls (Fig. 6e). Immunohistochemistry also certified the low expression level of CUL7 in sh-CUL7 cells (Fig. 6f-g). In addition, the positive rate of Ki-67 was decreased in sh-CUL7 xenografts (Fig. 6f-g). Interestingly, MST1 expression was significantly increased in the sh-CUL7 group compared with the control group. In contrast, the expression of N-cadherin was significantly decreased in the sh-CUL7 group (Fig. 6f-g). These results demonstrated that CUL7 silencing led to reduced proliferation and invasion of glioma cells in vivo.

### CUL7 is a direct target of miR-3940-5p

**Fig. 7:**
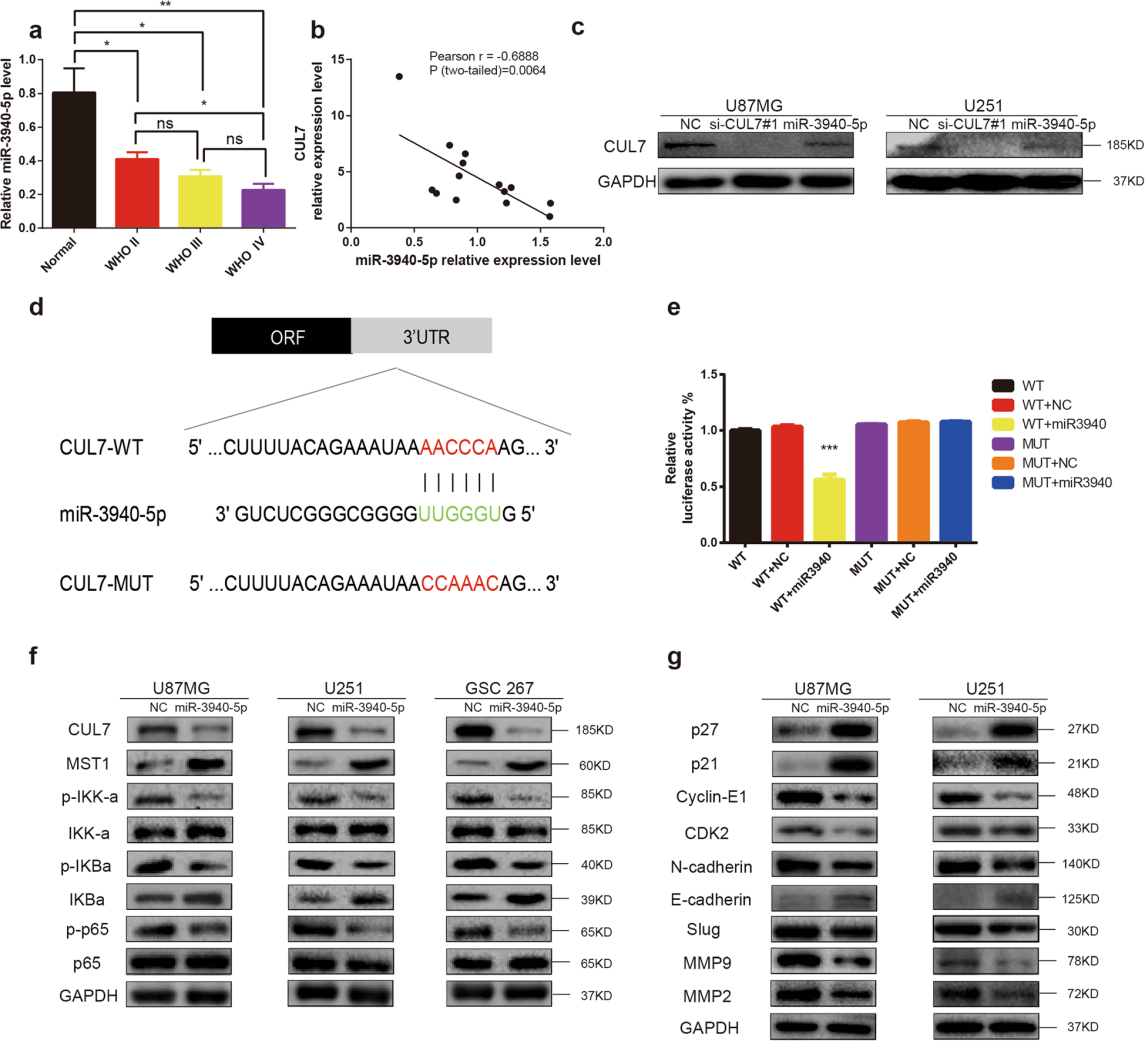
CUL7 is a direct target of miR-3940-5p in glioma cells**. a** qRT-PCR analysis validated the lower expression of miR-3940-5p in glioma tissues. **b** Pearson r correlation was used to analyze the relationship between CUL7 and miR-3940-5p in Qilu hospital patient samples. (*n* = 14). **c** Overexpression of miR-3940-5p markedly suppressed the protein levels of CUL7 in glioma cells. **d** miR-3940-5p and its putative binding sequence in the wild-type (WT) and mutant (MUT) 3′-UTR of CUL7. **e** Overexpression of miR-3940-5p significantly decreased the luciferase activity that carried wild-type (WT) but not mutant type (MUT) 3′-UTR of CUL7 in glioma cells. **f** Western blot to detect expression levels of the MST1 and markers of activation of NF-κB pathway. GAPDH was used as a loading control. **g** Western blot for protein levels of cell cycle regulatory factors and EMT components in lysates (20 μg) from U87MG and U251 cells transfected with miR-3940-5p mimics and controls. GAPDH was used as a loading control. *P < 0.05; **P < 0.01; ***P < 0.001; NC: negative control RNA; si-CUL7: siRNAs targeting CUL7; miR-3940-5p: miR-3940-5p mimics

To explore the potential miRNAs regulating CUL7, we first used the online miRNA target prediction algorithm TargetScan. We found that miR-3940-5p was highly likely to directly target CUL7. QRT-PCR demonstrated that miR-3940-5p was significantly downregulated in glioma cell lines compared with NHA (Fig. S4a), which predicted that miR-3940-5p was a tumour suppressor. Moreover, the miR-3940-5p expression level was significantly lower in glioma than in normal brain, which was opposite to the trend in CUL7 expression level (Fig. 7a-b). Interestingly, qRT-PCR and western blotting demonstrated that overexpression of miR-3940-5p (via miR-3940-5p mimics transfected into cell lines after 48 h) induced a strong decrease in CUL7 expression in glioma cell lines (Fig. S4b-c, Fig. 7c). To further validate the direct interactions between CUL7 and miR-3940-5p, we performed luciferase reporter assays. A vector encoding the wild-type (WT) sequence of the 3′-UTR of CUL7 mRNA or a vector encoding the mutant (MUT) sequence of the 3′-UTR of CUL7 mRNA lacking the predicted miR-3940-5p target site were transfected into 293 T cells. The putative target site of miR-3940-5p in the 3′-UTR of CUL7 is illustrated in Fig. 7d. The relative luciferase activity of the WT construct of the CUL7 3′-UTR in miR-3940-5p-transfected cells decreased to approximately 40% compared with that of the control miRNA, but the MUT construct of the CUL7 3′-UTR abolished the suppressive effect of miR-3940-5p (Fig. 7e). The above results showed that CUL7 is a direct target of miR-3940-5p.

### MiR-3940-5p inhibits the proliferation, migration and invasion of glioma cells

**Fig. 8:**
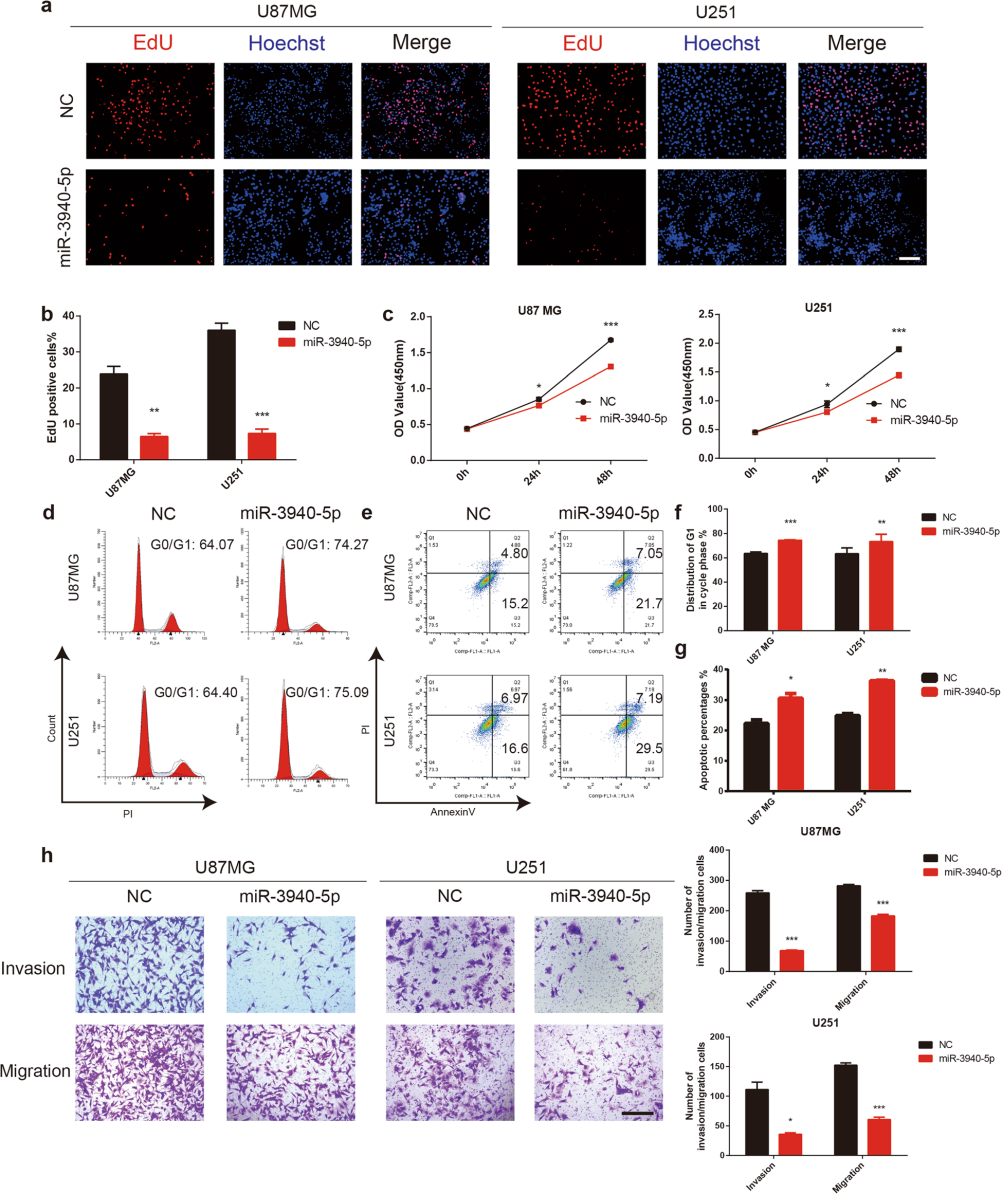
MiR-3940-5p inhibits cell proliferation, migration and invasion of glioma**.** U87MG and U251 cells transfected with miR-3940-5p mimics or controls and characterized in the following assays: (**a, b**) EdU performed 48 h after transfection (scale bar = 100 μm); (**c**) growth curve based on OD450 using the CCK-8 assay; (**d, f**) cell cycle profiles determined from PI staining in flow cytometry (**e, g**) % apoptosis as determined with Annexin V-FITC antibody and PI staining in flow cytometry. (**h**) Representative images of transwell migration and invasion assays performed in U87MG and U251 transfected NC and miR-3940-5p cells. Graphic representation of migrated and invaded cells counts from transwell assay (Scale bar = 200 μm). Data are represented as the mean ± SEM from three independent experiments. *P < 0.05; **P < 0.01; ***P < 0.001, relative to control. NC: negative control RNA; miR-3940-5p: miR-3940-5p mimics

To further investigate the biological roles of miR-3940-5p in gliomas, EdU assays, CCK-8 assays, flow cytometry analysis, colony forming assays and Transwell chamber assays were conducted with the U87MG and U251 cell lines that were transfected with miR-3940-5p mimics or miR-NC. The results showed that the proliferation, migration and invasion of glioma cells were notably suppressed in the miR-3940-5p mimic group (Fig. 8a-h, Fig. S4d-e). The prominent effect of miR-3940-5p overexpression on the self-renewal abilities of GSCs was verified by a tumoursphere formation assay (Fig. S4f-g). Moreover, the results of western blotting revealed that the changes in the expression levels of MST1, NF-κB signalling pathway markers, cyclin-dependent kinase inhibitors, cyclin-dependent kinase, and EMT markers in the cells transfected with miR-3940-5p mimics were the same as those in the CUL7-silenced cells (Fig. 7f-g).

To further confirm that miR-3940-5p inhibits cell proliferation, migration and invasion of glioma by targeting CUL7, we restored CUL7 expression by transfecting U87MG and U251 glioma cells with full-length CUL7 plasmids. The EdU assay showed that the effects of miR-3940-5p on glioma proliferation were counteracted by CUL7 overexpression (Fig. S5a-b). These findings were further confirmed by CCK-8 assays (Fig. S5c). Cell cycle analysis also demonstrated that overexpression of CUL7 offset the effects of miR-3940-5p on the population of U87MG and U251 cells in G0/G1 phase (Fig. S5d-e). In addition, overexpression of CUL7 inhibited the apoptosis of the U87MG and U251 cell lines induced by miR-3940-5p (Fig. S5f-g). Furthermore, Transwell assays showed that the effects of miR-3940-5p on glioma migration and invasion were counteracted by CUL7 overexpression (Fig. S6a-b). Moreover, western blot analysis confirmed that CUL7 overexpression attenuated the effects of miR-3940-5p (Fig. S6c-d). These results indicated that miR-3940-5p inhibits glioma tumorigenesis by downregulating CUL7.

## Discussion

At present, the prognosis of glioma patients is very poor, even when multimodal treatment strategies are used. Many important efforts have been made to identify prognostic molecular biomarkers that could provide explanations regarding glioma formation and progression. In this study, we showed that CUL7 was highly expressed in gliomas, especially in mesenchymal subtypes, and found that the expression of CUL7 increased as the overall survival of patients decreased, which demonstrated that CUL7 plays a significant role in the malignancy of glioma. In contrast, low CUL7 mRNA levels were linked to other positive prognostic markers, including IDH1 and ATRX mutations, MGMT methylation, 1p/19q codeletion, and loss of TERT expression. To test this finding, we verified that downregulation of CUL7 inhibited the proliferation, migration and invasion of glioma cell lines in vitro and in vivo.

Abnormal cell proliferation and growth are characteristics of human gliomas [39]. Many genetic changes induce uncontrolled growth through dysregulation of many proteins that are directly or indirectly involved in cell cycle progression and apoptosis [39]. GSEA analysis indicated that CUL7 might indeed promote proliferation and inhibit apoptosis, which was supported by in vitro and in vivo evidence. CUL7 silencing in glioma cells inhibited proliferation through cell cycle arrest at G0/G1 and induced cell apoptosis and reduced growth in orthotopic xenografts. The results show that knockdown of CUL7 leads to reduced cell proliferation in vitro, and in vivo data suggest that it is a potential molecular target for therapy. Moreover, CUL7 overexpression promoted proliferation in glioma cells in our study. Although many previous studies have reported that CUL7 promoted tumorigenesis by suppressing P53 [10, 40, 41], CUL7 also promoted the proliferation of U251, a glioma cell line with mutated p53 [42], in our study; this indicates that CUL7 could have other independent pathways to promote the development of glioma. In this way, to attain the potential molecular mechanisms by which CUL7 promotes glioma development, we detected the expression changes of some key mediators of cell proliferation and apoptosis, including p21, p27, cyclin D1, CDK4, cyclin E1 and CDK2. In our study, CUL7 knockdown led to significantly increased levels of tumour suppressors p21 and p27, resulting in the reduction of downstream oncogenic factors, including cyclin D1, CDK4, cyclin E1 and CDK2. In contrast, CUL7 overexpression decreased the levels of tumour suppressors and upregulated downstream oncogenic factors. These data suggest that CUL7 promotes cell proliferation in gliomas.

EMT is a key event driving the invasion of glioma cells [43, 44]. In addition, several EMT-related factors are associated with increased invasion and poor prognosis in gliomas [45–47]. Here, we observed that CUL7 depletion significantly reversed EMT features and decreased invasiveness in glioma cells. Using western blotting, we observed that several mesenchymal markers, such as N-cadherin, Vimentin and Slug, were decreased and that an epithelial factor (E-cadherin) was increased after CUL7 depletion in gliomas. CUL7 silencing appeared to specifically suppress the progression of EMT in glioma cells, which reduced their invasion abilities. Moreover, in our study, CUL7 overexpression promoted the progression of EMT in glioma cells. Therefore, these results indicated that CUL7 may serve as a crucial regulator of invasion and migration by inducing mesenchymal-like properties in gliomas.

The NF-κB pathway regulates gliomagenesis [48, 49]. In cells, IκB interacts with NF-κB(p65), leading to NF-κB(p65)/IκB complex sequestration in the cytoplasm, preventing NF-κB(p65) from binding to target DNA sequences. Some signalling cascades activate IKK, and IKK phosphorylates IκB in the cytoplasm, resulting in IκB degradation by the proteasome and NF-κB (p65) release from the inhibitory complex. Then, NF-κB proteins translocate into the nucleus, where they bind to DNA and activate gene transcription [50]. Lee et al. established that MST1 negatively regulates the NF-κB pathway by inhibiting the E3 ligase activity of HOIP and acts as a component of TNF-RSC that inhibits the linear ubiquitin chain-forming activity of LUBAC in a TRAF2-dependent manner [17]. We found that CUL7 activated the NF-κB pathway by promoting ubiquitin-mediated MST1 protein degradation using coimmunoprecipitation (co-IP) and western blot analysis.

Consistent with our current study, CUL7 has been reported to be upregulated and to induce oncogenic functions in multiple cancers. For example, CUL7 promotes the survival of a variety of cancer cells, including MDA-MB-231 breast cancer cells, HeLa cells and HEK293T cells, by promoting caspase-8 ubiquitination [51]. In addition, CUL7 overexpression and unfavourable prognosis have been reported to be correlated in hepatocellular carcinoma [11], epithelial ovarian cancer [12], lung cancer [13], breast cancer [14], and choriocarcinoma [15]. Thus, CUL7 may be a broad-spectrum biomarker and therapeutic target.

Although we analysed only two cell lines (U87MG and U251) and one glioma stem cell line (GSC267) in these studies, our orthotopic xenograft results provide powerful evidence; therefore, we speculate that other glioma cell lines will show similar results.

The role of CUL7 and its related pathways have been well demonstrated, but the upstream regulators of CUL7 remain largely unknown. miRNAs play a critical role in the posttranscriptional or translational regulation of gene expression [52]. To date, no studies have focused on the miRNAs regulating CUL7. In this regard, we conducted searches to identify miRNAs that might regulate CUL7 based on bioinformatic analysis and found that miR-3940-5p negatively regulated the expression of CUL7 in glioma cells. Recently, it has been reported that miR-3940-5p expression of miR-3940-5p is reduced in non-small-cell lung carcinoma and functions as a tumour suppressor [53, 54]. However, while one miRNA can target many genes, each gene can be regulated by multiple miRNAs [55]. In this way, further works are needed to discover the mechanisms involved in regulating CUL7.

## Conclusion

In conclusion, our study highlighted that increased CUL7 expression levels were associated with a higher tumour grade, a mesenchymal subtype and a poor prognosis in human gliomas. CUL7 facilitated the proliferation, invasion and migration of glioma cells by influencing MST1 ubiquitination and activation of NF-κB pathways. Additionally, we identified that CUL7 is a direct target of miR-3940-5p and that there is a suppressive role of miR-3940-5p in proliferation, invasion and migration in glioma cells. Collectively, the miR-3940-5p/CUL7/ NF-κB pathway may be a novel candidate therapeutic target in glioma treatment.

## Supplementary information

**Additional file 1: Figure S1.** Expression of CUL7 is associated with tumor grade and patient survival in gliomas. a Quantification of CUL7 mRNA expression levels in gliomas in CGGA. b Quantification of gliomas subtype-specific CUL7 expression in CGGA. Log2-transformed expression of CUL7 mRNA levels are listed on the Y-axis. Error bars represents the SEM. c IHC results presented the increase of CUL7 protein in high grade glioma. (WHO I-II; *n* = 17; WHO III-IV; *n* = 21; and normal brain tissues; *n* = 4) d CUL7 levels normalized to GAPDH in gliomas. (WHO II; *n* = 3; WHO III-IV; *n* = 8; and normal brain tissues; n = 3) e, f Kaplan–Meier survival analysis for glioma patients with high CUL7 expression and low CUL7 expression in LGGs (*n* = 165) or GBMs (*n* = 120) in CGGA database. The cut-off level was set at the median value of the CUL7 levels. *P < 0.05; **P < 0.01; ***P < 0.001.

**Additional file 2: Figure S2.** The results of GSEA analysis of CUL7 and quantitative and statistical analysis of MST1 expression level in CHX chase. a-e GSEA highlighting positive association of increased CUL7 expression levels with cancer metastasis, apoptosis, cell proliferation, EMT and Phillips-queried MES/PN gene set. NES = normalized enrichment score; NOM = nominal FDR = false discovery rate. f Line graph shows MST1 levels normalized to GAPDH at the indicated time points (n = 4). Data are represented as the mean ± SEM.

**Additional file 3: Figure S3.** CUL7 knockdown inhibits colony forming in U87MG and U251 cells and tumorsphere formation in GSC267 cells. CUL7 overexpression promoted the activation of NF-κB pathway. MST1 protein levels were downregulated in high grade and mesenchymal subtype glioma tissues. a, b cells were fixed and stained, colonies were counted, and results are represented in the bar graph(*n* = 3). Data are represented as the mean ± SEM from three independent experiments. c, d Tumorsphere formation assay of GSC267. Representative images of GSC tumorspheres (left panels, scale bar 100 μm; n = 3), the quantification of numbers (right panels) of the GSC tumorspheres are shown here. Data are shown as the mean ± SEM, *P < 0.05; **P < 0.01; ***P < 0.001, relative to control.NC: negative control RNA e Western blot for protein levels of cell cycle regulatory factors and EMT components in lysates (20 μg) from U87MG and U251 cells transfected with CUL7 and Vector. GAPDH was used as a loading control. f Western blot to detect expression levels of the MST1 and markers of activation of NF-κB pathway. GAPDH was used as a loading control. g Western blot for protein levels of CUL7 in lysates (20 μg) from U87MG and U251 cells transfected with shRNA against CUL7 and controls. h Representative images of IHC staining for MST1 in gliomas of different grades, mesenchymal subtype gliomas and non-mesenchymal subtype glioma tissues (scale bar = 100 μm).

**Additional file 4: Figure S4.** MiR-3940-5p decreases CUL7 mRNA level and inhibits colony forming in U87MG and U251 cells and tumorsphere formation in GSC267 cells. a qRT-PCR analysis validated the lower expression of miR-3940-5p in glioma. b, c qRT-PCR showed overexpression of miR-3940-5p markedly suppressed the protein levels of CUL7 in glioma cells. d, e cells were fixed and stained, colonies were counted, and results are represented in the bar graph. Data are represented as the mean ± SEM from three independent experiments. f, g Tumorsphere formation assay of GSC267. Representative images of GSC tumorspheres (left panels, scale bar 100 μm), the quantification of numbers (right panels) of the GSC tumorspheres are shown here. Data are shown as the mean ± SEM, n = 3 *P < 0.05; **P < 0.01; ***P < 0.001, relative to control.NC: negative control RNA.

**Additional file 5: Figure S5.** The effects of miR-3940-5p on proliferation of glioma were counteracted by CUL7 overexpression. U87MG and U251 cells transfected with miR-3940-5p mimics or controls and restored CUL7, and characterized in the following assays: (a, b) EdU performed 48 h after transfection (scale bar = 100 μm); (c) growth curve based on OD450 using the CCK-8 assay; (d, e) cell cycle profiles determined from PI staining in flow cytometry (f, g) % apoptosis as determined with Annexin V-FITC antibody and PI staining in flow cytometry. *P < 0.05; **P < 0.01; ***P < 0.001. NC: negative control RNA; miR-3940-5p: miR-3940-5p mimics; Vector: GV141-empty; CUL7: GV141-CUL7.

**Additional file 6: Figure S6.** The effects of miR-3940-5p on migration, invasion and activation of NF-κB pathway of glioma were counteracted by CUL7 overexpression. a, b Representative images of Transwell migration and invasion assays performed in U87MG and U251. Graphic representation of migrated and invaded cells counts from Transwell assay (Scale bar = 200 μm). Data are represented as the mean ± SEM from three independent experiments. *P < 0.05; **P < 0.01; ***P < 0.001, *n* = 3. c Western blot for protein levels of cell cycle regulatory factors and EMT components in lysates (20 μg) from U87MG and U251 cells. GAPDH was used as a loading control. d Western blot to detect expression levels of the MST1 and markers of activation of NF-κB pathway. GAPDH was used as a loading control. NC: negative control RNA; miR-3940-5p: miR-3940-5p mimics; Vector: GV141-empty; CUL7: GV141-CUL7.

## Data Availability

The dataset supporting the conclusions of this article was retrieved by using the TCGA, [http://cancergenome.nih.gov] and CGGA, [http://www.cgcg.org. cn/].

## Abbreviations

3’UTRs: 3′-untranslated regions
ATRX: Alpha thalassemia retardation syndrome X-linked
CCK-8: Cell Counting Kit-8
CDK2: Cyclin-dependent kinase 2
CDK4: Cyclin-dependent kinase 4
CGGA: The Chinese Glioma Genome Atlas
CUL7: Cullin-7
ECM: Extracellular matrix
EdU: 5-ethynyl-2′-deoxyuridine
EMT: Epithelial-mesenchymal transition
GBM: Glioblastoma multiforme
GSEA: Gene set enrichment analysis
IDH: Isocitrate dehydrogenase
IHC: Immunohistochemistry
LGG: Low grade glioma
MGMT: O-methylguanine-DNA methylguanine methyltransferase
miR: microRNA
MMP2: Matrix metalloproteinase 2
MMP9: Matrix metalloproteinase 9
OS: Overall survival
TCGA: The Cancer Genome Atlas
TERT: Telomerase reverse transcriptase
WHO: World Health Organization
