# Supplementary figures and images for: Cullin-7 (CUL7) is overexpressed in glioma cells and promotes tumorigenesis via NF-κB activation
Source: J Exp Clin Cancer Res. 2020 Apr 6;39:59. doi: 10.1186/s13046-020-01553-7 (PMC7132976; doi:10.1186/s13046-020-01553-7)

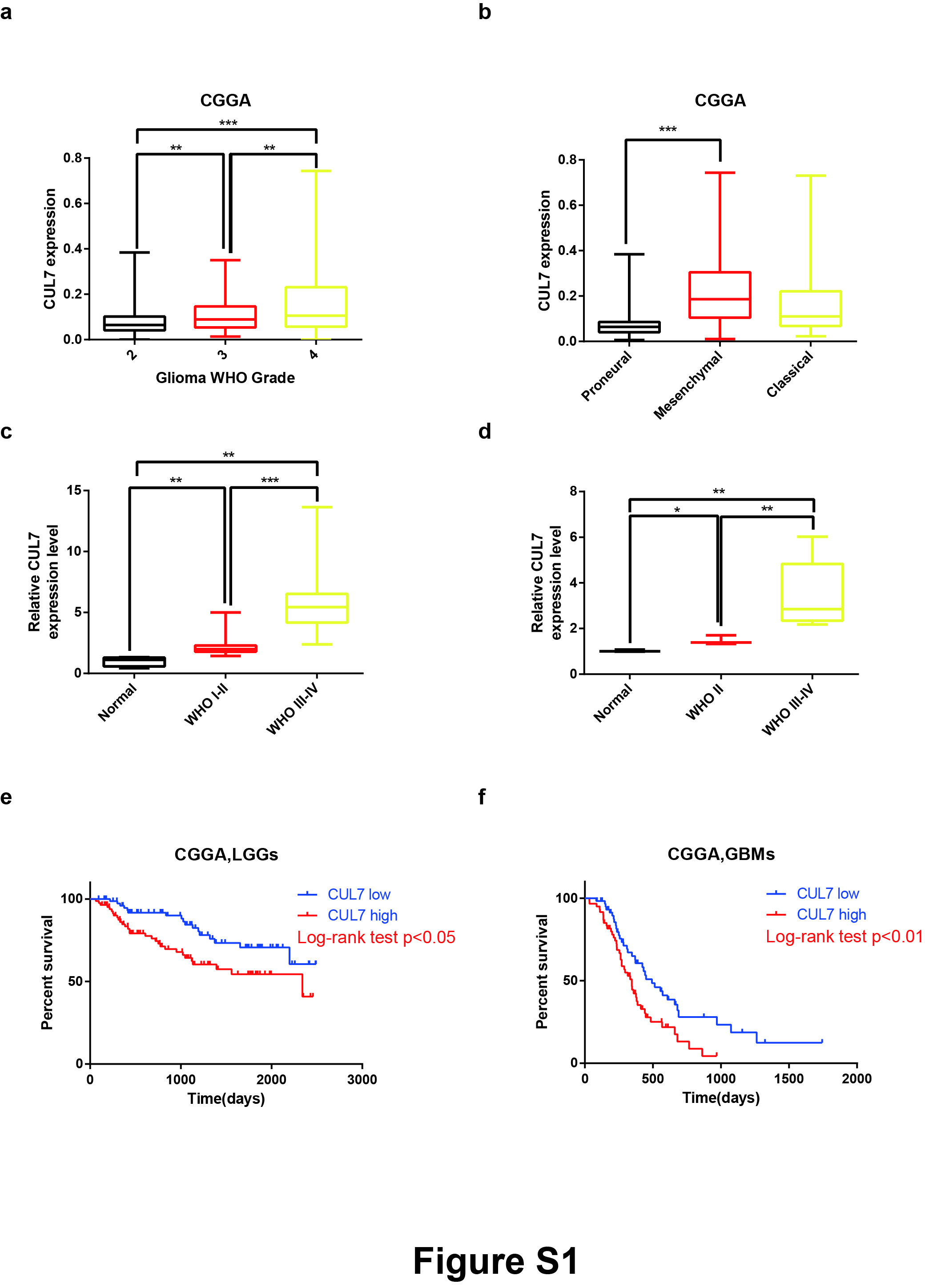

Supplement: Supplementary file 1 — Additional file 1: Figure S1. Expression of CUL7 is associated with tumor grade and patient survival in gliomas. a Quantification of CUL7 mRNA expression levels in gliomas in CGGA. b Quantification of gliomas subtype-specific CUL7 expression in CGGA. Log2-transformed expression of CUL7 mRNA levels are listed on the Y-axis. Error bars represents the SEM. c IHC results presented the increase of CUL7 protein in high grade glioma. (WHO I-II; n = 17; WHO III-IV; n = 21; and normal brain tissues; n = 4) d CUL7 levels normalized to GAPDH in gliomas. (WHO II; n = 3; WHO III-IV; n = 8; and normal brain tissues; n = 3) e, f Kaplan–Meier survival analysis for glioma patients with high CUL7 expression and low CUL7 expression in LGGs (n = 165) or GBMs (n = 120) in CGGA database. The cut-off level was set at the median value of the CUL7 levels. *P < 0.05; **P < 0.01; ***P < 0.001. [file 13046_2020_1553_MOESM1_ESM.tif]

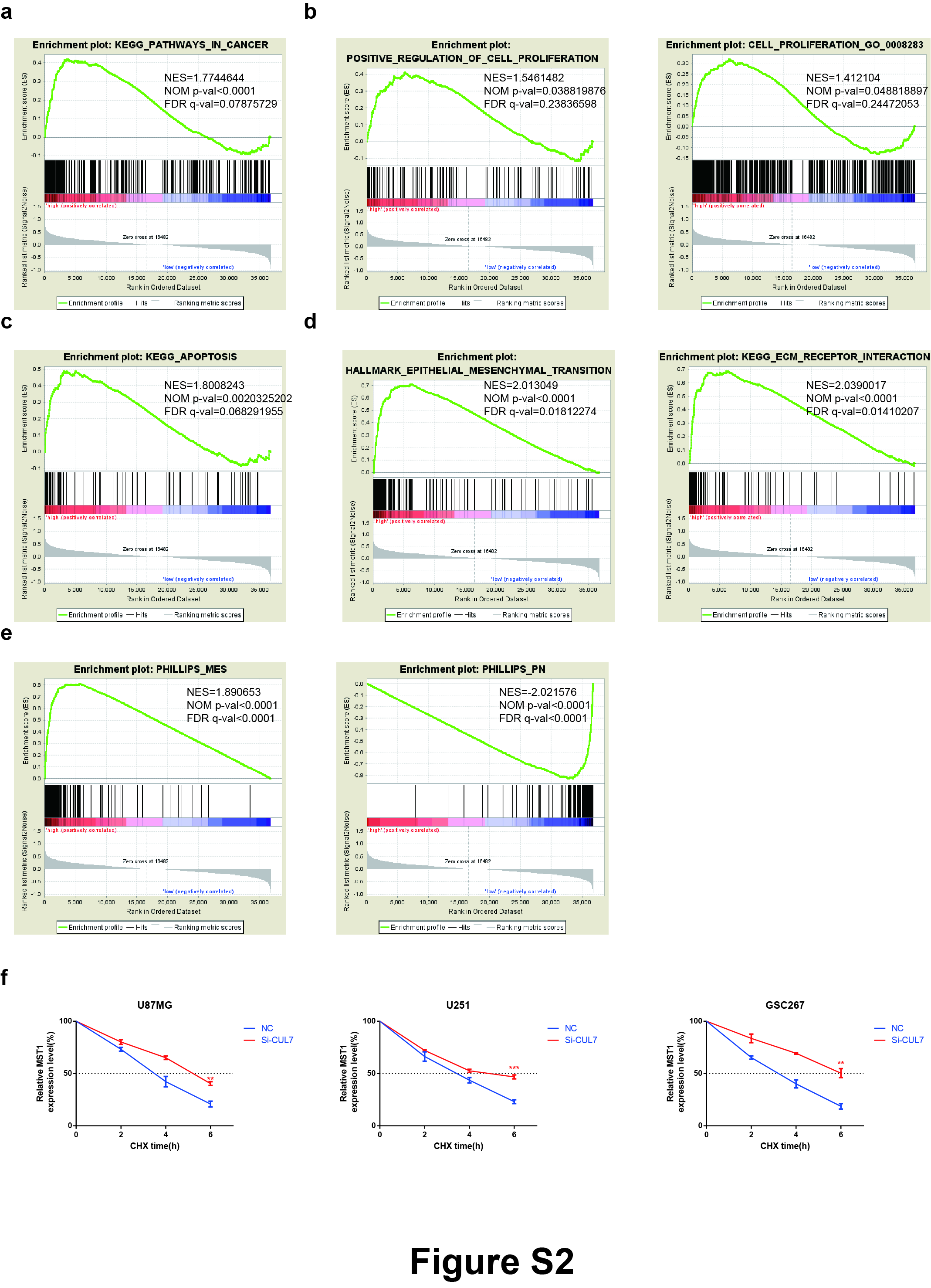

Supplement: Supplementary file 2 — Additional file 2: Figure S2. The results of GSEA analysis of CUL7 and quantitative and statistical analysis of MST1 expression level in CHX chase. a-e GSEA highlighting positive association of increased CUL7 expression levels with cancer metastasis, apoptosis, cell proliferation, EMT and Phillips-queried MES/PN gene set. NES = normalized enrichment score; NOM = nominal FDR = false discovery rate. f Line graph shows MST1 levels normalized to GAPDH at the indicated time points (n = 4). Data are represented as the mean ± SEM. [file 13046_2020_1553_MOESM2_ESM.tif]

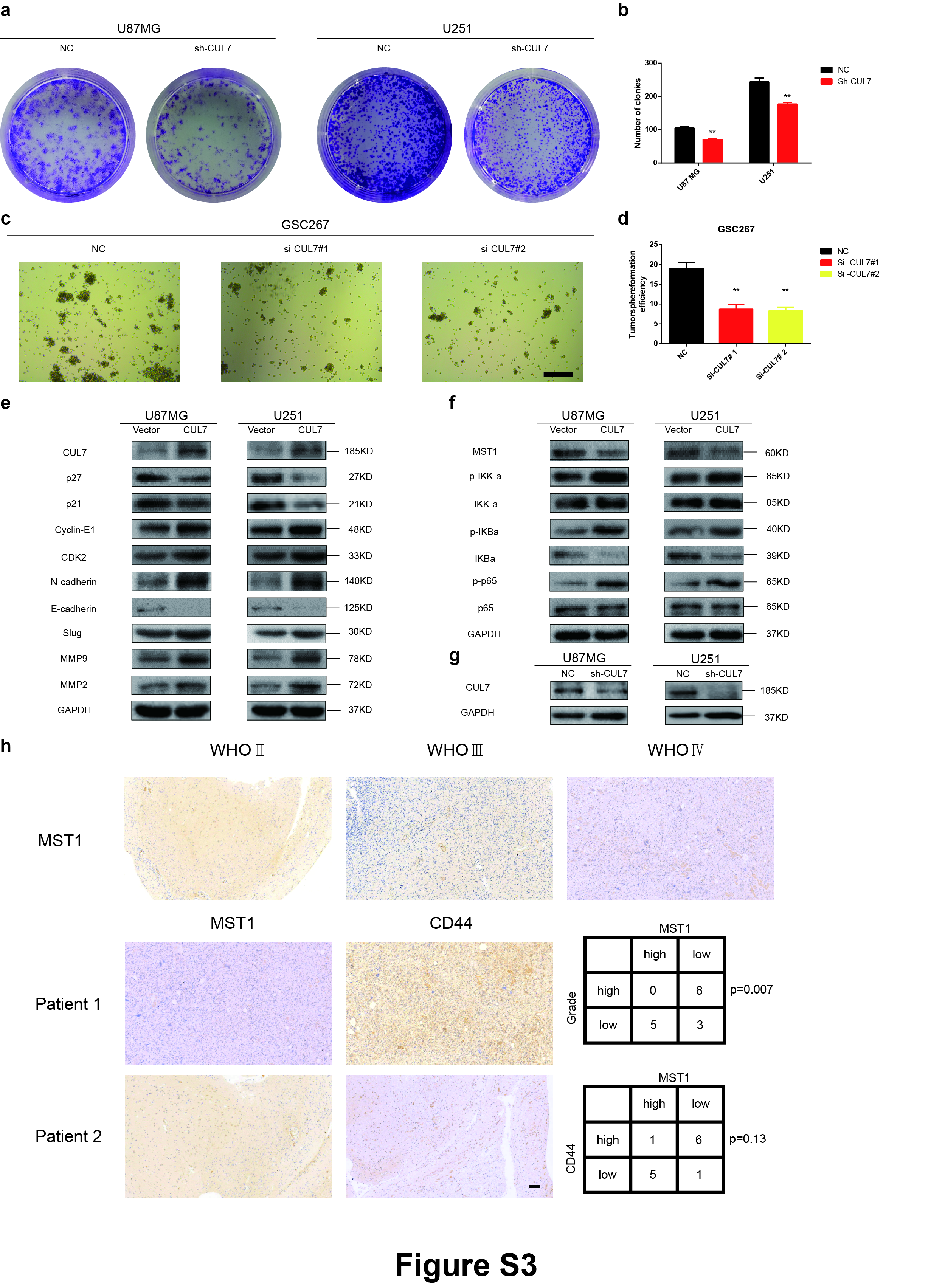

Supplement: Supplementary file 3 — Additional file 3: Figure S3. CUL7 knockdown inhibits colony forming in U87MG and U251 cells and tumorsphere formation in GSC267 cells. CUL7 overexpression promoted the activation of NF-κB pathway. MST1 protein levels were downregulated in high grade and mesenchymal subtype glioma tissues. a, b cells were fixed and stained, colonies were counted, and results are represented in the bar graph(n = 3). Data are represented as the mean ± SEM from three independent experiments. c, d Tumorsphere formation assay of GSC267. Representative images of GSC tumorspheres (left panels, scale bar 100 μm; n = 3), the quantification of numbers (right panels) of the GSC tumorspheres are shown here. Data are shown as the mean ± SEM, *P < 0.05; **P < 0.01; ***P < 0.001, relative to control.NC: negative control RNA e Western blot for protein levels of cell cycle regulatory factors and EMT components in lysates (20 μg) from U87MG and U251 cells transfected with CUL7 and Vector. GAPDH was used as a loading control. f Western blot to detect expression levels of the MST1 and markers of activation of NF-κB pathway. GAPDH was used as a loading control. g Western blot for protein levels of CUL7 in lysates (20 μg) from U87MG and U251 cells transfected with shRNA against CUL7 and controls. h Representative images of IHC staining for MST1 in gliomas of different grades, mesenchymal subtype gliomas and non-mesenchymal subtype glioma tissues (scale bar = 100 μm). [file 13046_2020_1553_MOESM3_ESM.tif]

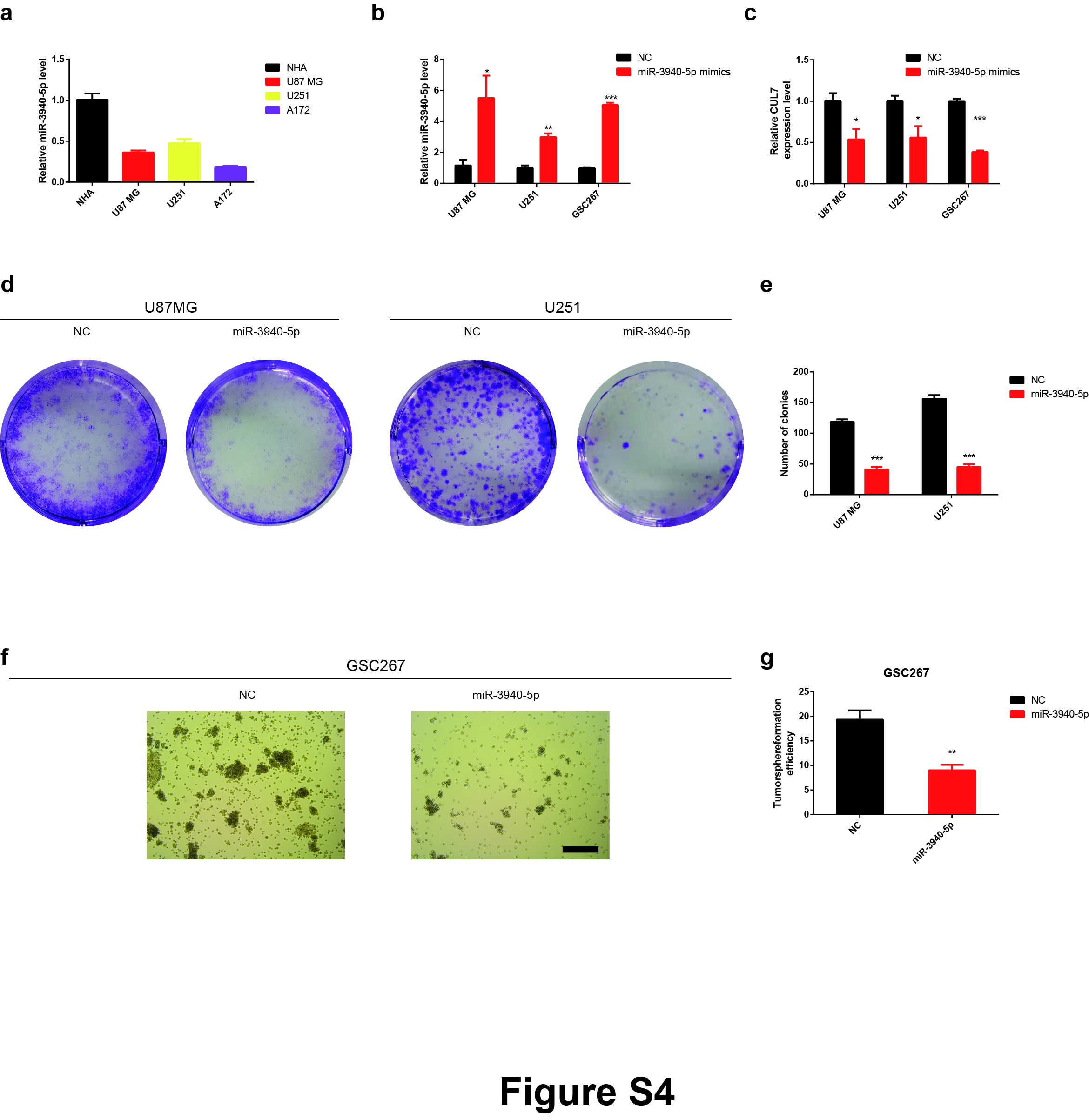

Supplement: Supplementary file 4 — Additional file 4: Figure S4. MiR-3940-5p decreases CUL7 mRNA level and inhibits colony forming in U87MG and U251 cells and tumorsphere formation in GSC267 cells. a qRT-PCR analysis validated the lower expression of miR-3940-5p in glioma. b, c qRT-PCR showed overexpression of miR-3940-5p markedly suppressed the protein levels of CUL7 in glioma cells. d, e cells were fixed and stained, colonies were counted, and results are represented in the bar graph. Data are represented as the mean ± SEM from three independent experiments. f, g Tumorsphere formation assay of GSC267. Representative images of GSC tumorspheres (left panels, scale bar 100 μm), the quantification of numbers (right panels) of the GSC tumorspheres are shown here. Data are shown as the mean ± SEM, n = 3 *P < 0.05; **P < 0.01; ***P < 0.001, relative to control.NC: negative control RNA. [file 13046_2020_1553_MOESM4_ESM.tif]

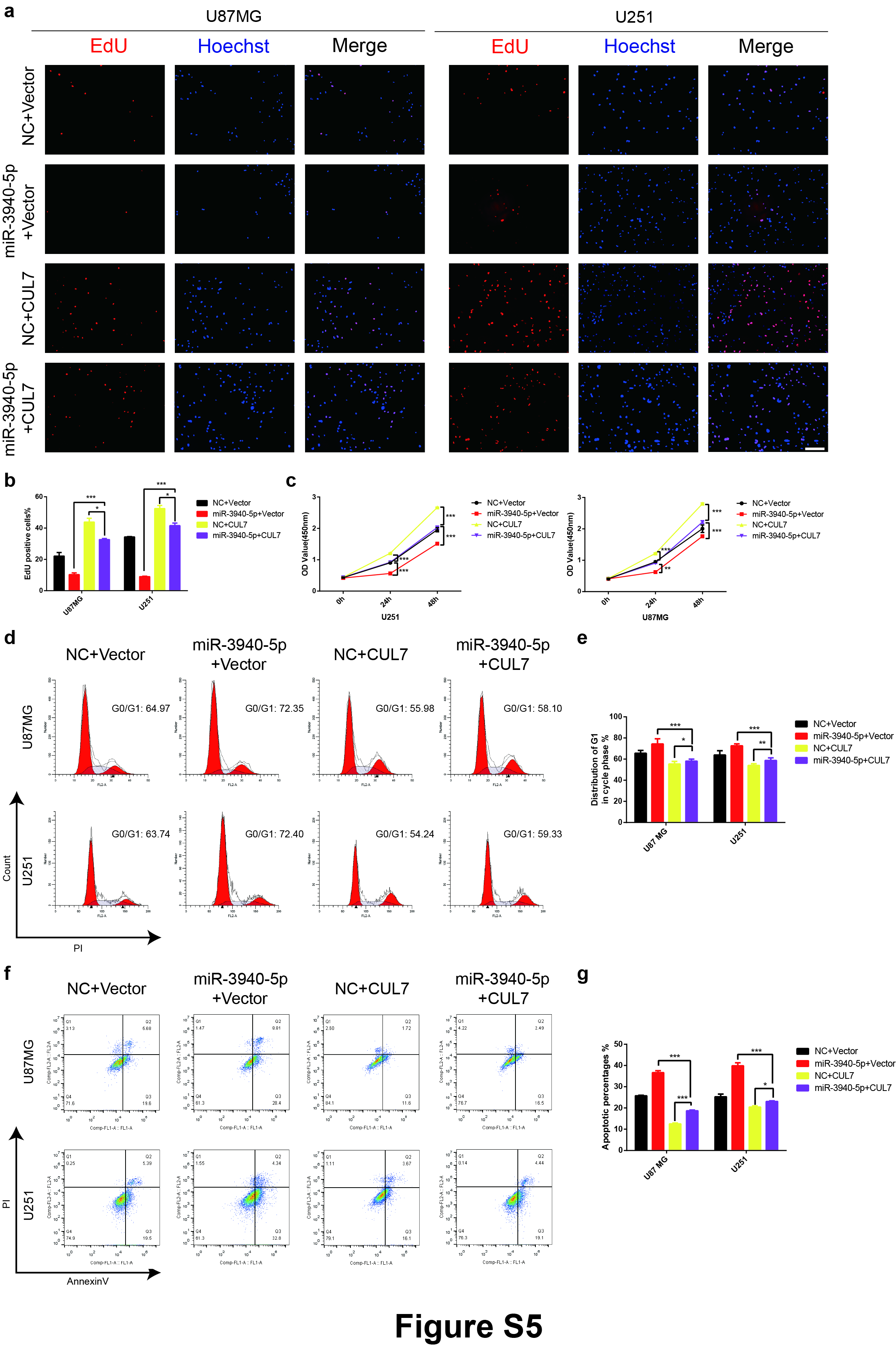

Supplement: Supplementary file 5 — Additional file 5: Figure S5. The effects of miR-3940-5p on proliferation of glioma were counteracted by CUL7 overexpression. U87MG and U251 cells transfected with miR-3940-5p mimics or controls and restored CUL7, and characterized in the following assays: (a, b) EdU performed 48 h after transfection (scale bar = 100 μm); (c) growth curve based on OD450 using the CCK-8 assay; (d, e) cell cycle profiles determined from PI staining in flow cytometry (f, g) % apoptosis as determined with Annexin V-FITC antibody and PI staining in flow cytometry. *P < 0.05; **P < 0.01; ***P < 0.001. NC: negative control RNA; miR-3940-5p: miR-3940-5p mimics; Vector: GV141-empty; CUL7: GV141-CUL7. [file 13046_2020_1553_MOESM5_ESM.tif]

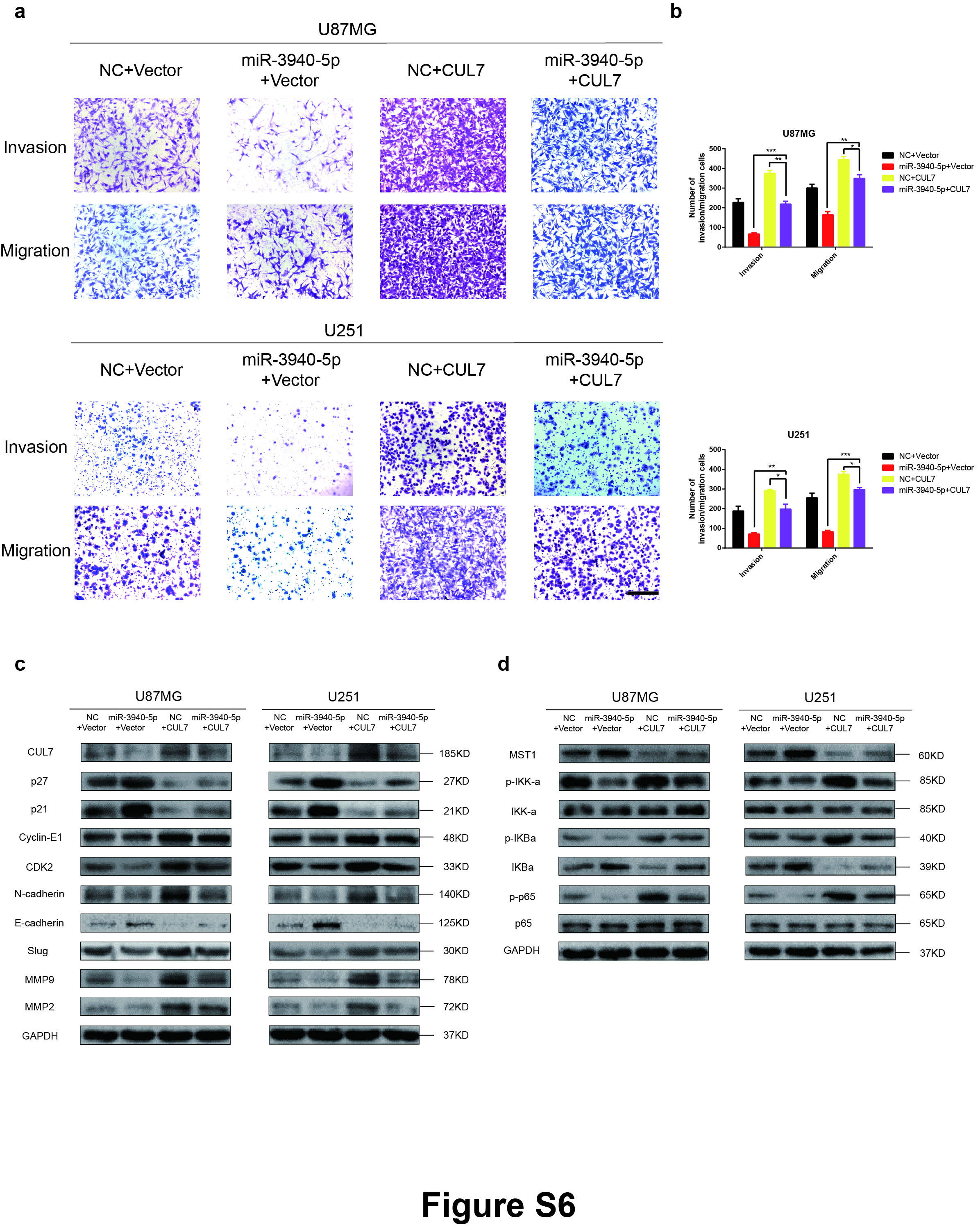

Supplement: Supplementary file 6 — Additional file 6: Figure S6. The effects of miR-3940-5p on migration, invasion and activation of NF-κB pathway of glioma were counteracted by CUL7 overexpression. a, b Representative images of Transwell migration and invasion assays performed in U87MG and U251. Graphic representation of migrated and invaded cells counts from Transwell assay (Scale bar = 200 μm). Data are represented as the mean ± SEM from three independent experiments. *P < 0.05; **P < 0.01; ***P < 0.001, n = 3. c Western blot for protein levels of cell cycle regulatory factors and EMT components in lysates (20 μg) from U87MG and U251 cells. GAPDH was used as a loading control. d Western blot to detect expression levels of the MST1 and markers of activation of NF-κB pathway. GAPDH was used as a loading control. NC: negative control RNA; miR-3940-5p: miR-3940-5p mimics; Vector: GV141-empty; CUL7: GV141-CUL7. [file 13046_2020_1553_MOESM6_ESM.tif]
